# Homeodomain-like DNA binding proteins control the haploid-to-diploid transition in *Dictyostelium*

**DOI:** 10.1126/sciadv.1602937

**Published:** 2017-09-01

**Authors:** Katy Hedgethorne, Sebastian Eustermann, Ji-Chun Yang, Tom E. H. Ogden, David Neuhaus, Gareth Bloomfield

**Affiliations:** MRC Laboratory of Molecular Biology, Francis Crick Avenue, Cambridge CB2 0QH, UK.

## Abstract

Homeodomain proteins control the developmental transition between the haploid and diploid phases in several eukaryotic lineages, but it is not known whether this regulatory mechanism reflects the ancestral condition or, instead, convergent evolution. We have characterized the mating-type locus of the amoebozoan *Dictyostelium discoideum*, which encodes two pairs of small proteins that determine the three mating types of this species; none of these proteins display recognizable homology to known families. We report that the nuclear magnetic resonance structures of two of them, MatA and MatB, contain helix-turn-helix folds flanked by largely disordered amino- and carboxyl-terminal tails. This fold closely resembles that of homeodomain transcription factors, and, like those proteins, MatA and MatB each bind DNA characteristically using the third helix of their folded domains. By constructing chimeric versions containing parts of MatA and MatB, we demonstrate that the carboxyl-terminal tail, not the central DNA binding motif, confers mating specificity, providing mechanistic insight into how a third mating type might have originated. Finally, we show that these homeodomain-like proteins specify zygote function: Hemizygous diploids, formed in crosses between a wild-type strain and a *mat* null mutant, grow and differentiate identically to haploids. We propose that *Dictyostelium* MatA and MatB are divergent homeodomain proteins with a conserved function in triggering the haploid-to-diploid transition that can be traced back to the last common ancestor of eukaryotes.

## Teaser

Structures of social amoeba mating-type proteins suggest deep conservation of sexual differentiation processes.

## INTRODUCTION

Eukaryotic organisms have a wide variety of life cycles. Many are sexual, undergoing cycling between different ploidy levels, and even asexual species change their ploidy, although with less regularity (*1*, *2*). Sexual life cycles are themselves highly diverse, because both haploid and diploid phases can be either proliferative or nonproliferative and unicellular or multicellular, and regular polyploidy is not uncommon (*1*). Within this diversity, some features are relatively common, if not universal. Because meiosis is conserved very widely across eukaryotic lineages, as are proteins involved in cellular and nuclear fusion, it is believed that these sexual processes, and the genes responsible for them, were already present in the last common ancestor of eukaryotes (*3*).

The transition from the haploid to the diploid phase appears to have some features beyond the mechanics of membrane fusion that are potentially widely conserved: In diverse organisms, homeodomain proteins are required to trigger zygote function (*4*). First established in saccharomycete yeasts and then in basidiomycetes, this function of specific homeodomain-containing transcription factors has more recently been found to be present in green algae and land plants (*5*–*10*). Typically, two different homeodomain proteins are separately expressed in each gamete class so that, upon cell fusion, new heterodimers can be formed, enabling distinct DNA binding properties in haploid and diploid cells (*6*). Although the conservation of this feature of the haploid-to-diploid transition in fungi and plants suggests that it might be ancestral, zygote development in animals does not appear to be controlled in the same way, and it remains to be established whether this role of homeodomain proteins is conserved in the less well-studied eukaryotic lineages (*4*).

Genes specifically involved in sexual processes tend to evolve rapidly, reflecting selection resulting from competition or antagonism between genes that underlie traits important during fertilization and sexual development (*11*–*13*). In some lineages, this evolutionary plasticity extends to the number of sexes (using this word in its broadest sense to include mating types): In ciliates, mycetozoan amoebae, and basidiomycete fungi, the number of mating types varies between species and is frequently greater than two (*14*–*17*). A number of factors are likely to affect the number of mating types in different populations (*18*), including the requirement to trigger zygote development (*19*). A new mating type must not only find and fuse with other types efficiently but also trigger the same zygotic response in all pairings, and it is possible that the mechanism used in some species might make the invasion of a new mating type impossible. However, the case of basidiomycetes mentioned above, in which many pairwise interactions between distinct homeodomain proteins can lead to correct zygote function, shows that this potential limitation is capable of being overcome. As mentioned earlier, these organisms often have more than two mating types and use homeobox genes in mating-type determination: Alleles of these genes must be different to trigger the zygotic response (*7*, *8*). In cases where the number of mating types has reverted to two, an expanded mating-type locus includes two of these homeobox genes (*17*, *20*, *21*).

The social amoebae, or dictyostelids, are another clade in which multiple sexes are common. Unlike haploid dictyostelid cells, zygotes feed cannibalistically on other amoebae of the same species, growing without cell division to form a dormant walled structure called a macrocyst (*22*). This large cell is thought to go through meiosis before haploid progeny resumes the mitotic cell cycle (*15*, *23*). The most widely studied social amoeba, *Dictyostelium discoideum*, has three mating types (*24*). Cells of any two mating types are able to fuse pairwise in a process dependent on a combination of cell-cell interactions and subsequently initiate zygotic development (*25*). The mating-type locus of this species encodes two pairs of gametologs that suffice to specify sexual compatibility: *matA* for type I, *matS* for type III, and both *matB* and *matC* for type II. The *matA* and *matB* genes are homologous, as are *matC* and *matS*, so type II appears to be a composite version of the other two mating types. However, although *matB* is genetically compatible with *matS*, and *matA* with *matC*, in specifying sexually compatible pairs, *matB* and *matC* are incompatible (*24*). We have suggested that the third mating type in this species arose after recombination between the two other versions of the *mat* locus, but it remains unclear how *matA*- and *matS*-like genes that ordinarily trigger sexual development when expressed in the same cell lost their compatibility to do so in the course of their evolution into *matB* and *matC* (*15*). None of the proteins encoded by these genes have recognizable sequence homology to known protein families or to any predicted domains. Furthermore, homologs are only detectable in closely related dictyostelid genomes, suggesting that these genes are rapidly evolving or that they represent novel sequences that arose recently (*15*).

We have taken a structural approach to gain insight into the molecular functions of these apparently novel genes. Solution nuclear magnetic resonance (NMR) structures that we report here reveal that MatA and MatB each have a core helix-turn-helix (HTH) motif resembling a homeodomain, with largely flexible N- and C-terminal extensions, and we further show that the C-terminal tail is the main determinant of MatA versus MatB activity. We present evidence that these proteins are transcription factors with conserved roles, controlling the transition between the *Dictyostelium* haploid and diploid phases.

## RESULTS

### The solution structures of MatA and MatB reveal homeodomain-like folds

To uncover the mechanism by which the Mat proteins control mating-type determination in *D. discoideum*, we set out to solve the structures of MatA and MatB, the 107–amino acid proteins encoded by the mating-type locus in type I and type II cells; the small size and hydrophilic nature of these proteins make NMR spectroscopy particularly suitable for this purpose. Both proteins are monomeric in solution, as assessed by multiangle light scattering (MALS), and maintain their structures over a broad concentration range, as assessed by circular dichroism (CD) spectroscopy (fig. S1).

**Fig. 1:**
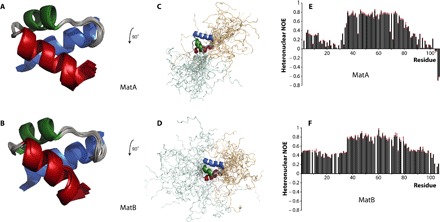
Solution structures of MatA and MatB determined by NMR spectroscopy. The folded core domains of MatA (**A**) and MatB (**B**) both contain three α helices arranged similarly to the homeodomain fold (helix 1 in blue, helix 2 in green, and helix 3 in red). The tail regions of MatA (**C**) and MatB (**D**) extend flexibly away from the well-folded core (N-terminal tails in pale cyan and C-terminal tails in light orange). Heteronuclear ^15^N{^1^H} NOE data for MatA (**E**) and MatB (**F**) suggest that, in both cases, there may be some structure in the disordered tails (see text). Relationships between the orientations of the different structural views are indicated on the figure; relative scalings of the views were chosen for clarity.

The NMR structure of MatA (Fig. 1, A and C; figs. S2 and S3; and table S1) shows that it consists of a well-ordered, folded core domain of approximately 50 amino acids arranged in three α helices (residues 36 to 50, 56 to 64, and 68 to 79) that pack together to form a structure resembling a homeodomain fold. This core domain is flanked by long, largely unstructured tails at both the N and C termini (Fig. 1C). The presence of a homeodomain-like fold suggests a function by which MatA controls mating-type determination in *D. discoideum*, namely, that it is a transcription factor that binds to DNA and acts as a master regulator of the gene expression pathways involved in sexual development.

The MatB protein encoded by the mating-type locus of type II shares 57% sequence identity with MatA but performs a distinct function. Expression of MatA alone in a *mat* null background leads to mating compatibility with type II and III strains, whereas cells expressing only MatB can mate only with type III strains (*24*). To search for structural differences between MatA and MatB that could be responsible for these different biological functions, we went on to determine the NMR structure of MatB. Unlike MatA, the MatB protein suffered from problems of thermal instability, and many signals in its NMR spectra exhibited poor line shapes and sensitivity (fig. S4), greatly hindering resonance assignment and structure determination. We tested whether truncation mutants (18 to 107, 18 to 86, and 31 to 86) that lack parts of the tails might alleviate these problems but found instead that they exacerbated them. However, making the single point mutation S71A and reducing the sample temperature to 1°C resulted in sufficient improvement to the quality of the NMR data (fig. S5) that the resonance assignment could be completed. Multiple alignments of the polypeptide sequences of *Dictyostelium* MatA and MatB homologs showed that MatB is the only example that does not have an alanine residue at this position in the second helix of the HTH motif; in addition, residue 71 bears one of the only three buried side chains that differ between *D. discoideum* MatA and MatB.

Despite these improvements, the NMR data for MatB S71A were still of much lower quality than those for MatA, making structure determination significantly more challenging and resulting in somewhat inferior structural statistics. Nonetheless, the ensemble structure of MatB (Fig. 1, B and D) shows that it has a very similar architecture to MatA: The core globular domain contains three α helices (residues 35 to 50, 57 to 64, and 68 to 79) again packed into a fold closely resembling the homeodomain and flanked by flexible N- and C-terminal tails.

We used steady-state ^15^N{^1^H} nuclear Overhauser effect (NOE) experiments to assess the flexibility of these tails in solution (Fig. 1, E and F). The plots for MatA and MatB share similar overall shapes, both showing high values of the NOE ratio for residues in a rigid core domain (approximately residues 35 to 80) and lower values in both N- and C-terminal tails. However, values for the tails are far from uniform; for both MatA and MatB, higher NOE ratios suggest that residual structure is present for residues 4 to 14, and the relatively slow decrease in NOE ratios along the C-terminal tail from the core suggests the possibility of some partial structure there, too. Analysis of secondary chemical shift data supports similar conclusions (fig. S6), with the tails of both MatA and MatB showing relatively small values of differences from random coil values, together with high but nonuniform values of the random coil index [a chemical shift–based indicator of backbone flexibility (*26*)]. These findings are consistent with the hypothesis that the N- and C-terminal tails of both MatA and MatB may fold upon interaction with partner proteins; it may be subtle differences between the structures formed by the tails during these interactions that specify their interaction partners and, hence, the different roles each protein plays in mating-type determination in *D. discoideum*.

### Structural and sequence homology suggest conserved functions for MatA and MatB

**Fig. 2:**
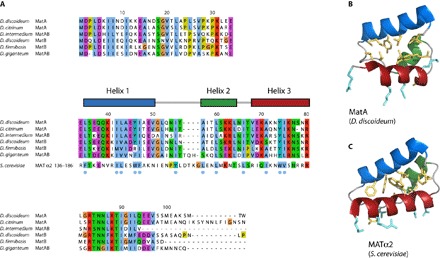
Sequence and structure-based alignments for MatA and MatB. (**A**) Sequence alignment of MatA and MatB homologs (CLUSTAL color scheme) from a variety of *Dictyostelium* species shows a high degree of conservation, implying that the structure is very likely to be conserved between these species. The separate lower row shown in the core region is a structure-based alignment of MatA with *S. cerevisiae* MATα2, which demonstrates that many of the core hydrophobic residues (indicated with blue dots) are also conserved between these two proteins. The structures of MatA (**B**) and MATα2 (**C**) show how, in both cases, the side chains of these conserved hydrophobic residues (shown in yellow) are arranged to form the core of the structure. Side chains of solvent-exposed basic residues on the third helix that are likely (MatA) or known (MATα2) to interact with the phosphate backbone of the DNA upon binding are shown in turquoise.

MatA and MatB homologs from various *Dictyostelium* species are highly divergent in sequence, and we have so far only been able to identify these homologs among dictyostelids closely related to *D. discoideum* (Fig. 2A). Despite this divergence, many of the hydrophobic residues within the core of the structured domain, and which are responsible for determining its fold, are largely conserved, suggesting that this core structure is likely to be conserved among the *Dictyostelium* MatA- and MatB-like proteins. To compare the *D. discoideum* MatA and MatB proteins with homeodomain proteins from other species, we first used FATCAT (*27*) to construct a structure-based alignment between the ordered domain of the MatA NMR structure and the crystal structure of the widely studied homeodomain protein MATα2 from *Saccharomyces* *cerevisiae*, a functional analog and possible distant homolog (Fig. 2A, middle) (*28*). This alignment shows that many of the hydrophobic residues considered to constitute the signature of the homeodomain (*29*) are conserved among these *D. discoideum* proteins. A comparison of the folded regions of MatA and MATα2 shows how several of these hydrophobic side chains pack between the three helices (Fig. 2, B and D). MatA and MatB have very low sequence similarity with established homeodomain proteins, even within the *D. discoideum* proteome (fig. S7), which is why sequence comparison alone was incapable of detecting these relationships. Certain bacterial and archaeal HTH proteins share a similar structure, for instance, the archaeal HTH-10 family, and we cannot yet confidently place MatA and MatB proteins within the homeodomain family in a phylogenetic tree (figs. S7 and S8). Nonetheless, considering the known roles for homeodomain proteins in controlling sexual cycles of other eukaryotes, we favor the hypothesis that MatA and MatB are divergent members of this family of transcription factors.

### MatA and MatB are DNA binding proteins

**Fig. 3:**
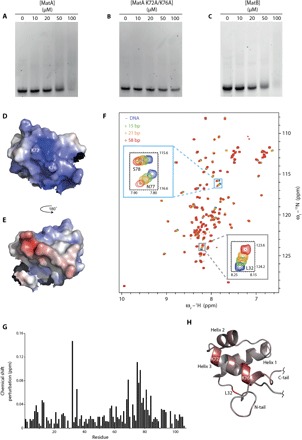
Assessing the DNA binding activity of MatA and MatB. (**A**) EMSA experiments show that adding an increasing concentration of MatA to a dsDNA oligonucleotide causes the free DNA band to be progressively replaced by a broad smear, indicating (nonspecific) binding (see text). (**B**) Mutating residues Lys^72^ and Lys^76^ to Ala abolishes this interaction, implicating these residues in the mechanism of DNA binding. (**C**) Addition of MatB causes a similar pattern to that seen for the addition of MatA. (**D** and **E**) Electrostatic potential surface of MatA, omitting the tail regions for clarity. The orientation shown in (D) (same as in Fig. 1A) shows the pronounced basic patch resulting from the conserved basic residues on the surface of helix 3. (**F**) Adding various lengths of dsDNA to samples of ^15^N-labeled MatA causes peaks to shift in the ^15^N-^1^H HSQC (heteronuclear single-quantum coherence) NMR spectrum; these CSPs can be plotted as a histogram (**G**) and mapped as a color ramp onto the lowest energy structure of MatA (**H**), shown in the same orientation as (D). This shows that many of the shifts map to the third helix, again implicating this region in direct interactions with the DNA; Leu^32^, which is N-terminal to the core folded domain, is also strongly affected. The NMR experiments used MatA (20 μM) and DNA (80 μM) in 25 mM phosphate (pH 6), 50 mM NaCl, and 50 μM EDTA. ppm, parts per million.

To determine whether, as their structures suggest, MatA and MatB could function as transcription factors, we assessed their DNA binding activity in vitro. To do this, we first performed an electrophoretic mobility shift assay (EMSA), adding increasing amounts of MatA to a sample of double-stranded DNA (dsDNA) (Fig. 3A). Because the sequence specificities of MatA and MatB are as yet unknown, we used a DNA oligonucleotide incorporating an operator sequence bound by *S. cerevisiae* MATα2 (*30*). Increasing the protein concentration causes the DNA band to smear, indicating that MatA does bind the oligonucleotide under these conditions but that the interaction is likely to be nonspecific and low affinity; this is not unexpected because this oligonucleotide probably does not contain the specific recognition sequence of MatA. Furthermore, it is possible that, like the yeast MATa1 protein, MatA could bind at high affinity only as part of a heterodimer (*31*). Mutation of residues Lys^72^ and Lys^76^ abrogates DNA binding (Fig. 3B), demonstrating that the DNA binding activity of MatA resides in the third helix (fig. S9), as has been shown for other homeodomain proteins (*28*); this is entirely consistent with the electrostatic potential surface shown in Fig. 3D, which shows this face of the homeodomain to be highly basic in character. Both wild-type MatB (Fig. 3C) and MatB S71A produce similar DNA band shifts to that observed for wild-type MatA (fig. S10), indicating that MatB also binds nonspecifically to the DNA sequence used in these experiments and that DNA binding is unaffected by the S71A mutation.

To further characterize the residues involved in DNA binding, we next turned to NMR measurements, monitoring amide group chemical shift perturbations (CSPs) resulting from addition of various lengths of dsDNA to ^15^N-labeled protein. In the case of MatB S71A at 1°C, addition of DNA resulted in disappearance of all signals except for the sharpest of those from the disordered tails. This was probably due to line broadening arising from intermediate rate exchange between free and bound states; the thermal instability of MatB S71A prevented the alleviation of this problem by raising the temperature. In contrast, in the case of MatA at 25°C, clear and localized effects were observed. Several peaks exhibited CSPs upon addition of DNA, and progressively larger CSPs were observed when longer DNA oligonucleotides were used, suggesting that avidity effects result in increased affinities as the DNA is lengthened (Fig. 3F). A titration experiment using the longest of these oligonucleotides (fig. S11) demonstrated that, as expected for a weakly interacting system, the system is in fast exchange on the NMR time scale (thus, all NMR properties represent population-weighted averages, with protein chemical shifts changing smoothly as a function of added DNA concentration). A group of the largest CSP values, including those for Glu^69^, Ile^75^, Lys^76^, and Asn^77^, map to the C-terminal helix of the MatA structure [Fig. 3, G and H; results shown for a 29–base pair (bp) oligonucleotide], reinforcing the results of the EMSA experiments with mutants that suggest that it is primarily this helix that interacts with DNA. Intriguingly, relatively large CSPs are also seen for Leu^32^ and Leu^35^, which are located in a boundary region between the N-terminal tail and the structured core. Although the possibility cannot be excluded that these CSPs might arise through DNA-induced conformational changes, it is noteworthy that several homeodomain proteins use residues N-terminal to the core homeodomain fold to make additional contacts with the DNA within the minor groove adjacent to the primary binding site (*29*, *32*); our results suggest that the same may well occur for MatA.

### The C-terminal tails functionally distinguish MatA and MatB

Many homeodomain proteins use flexible regions N- and C-terminal to the homeodomain fold to coordinate interactions with other proteins, often to increase binding affinity and sequence specificity (*32*), in addition to the N-terminal DNA contacts just mentioned. Considering the sequence divergence of the tails of MatA and MatB, it is possible that their different biological functions arise largely from differences in their tails, allowing them to interact with different partner proteins and produce distinctive patterns of gene expression.

**Fig. 4:**
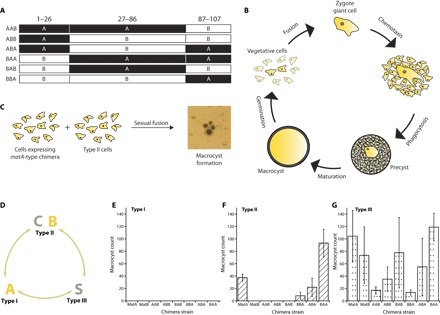
Using a macrocyst formation assay to assess the degree to which MatA/B chimeras exhibit MatA-type functionality. (**A**) A series of constructs were produced by exchanging the tails and core regions of MatA and MatB. (**B**) Vegetative *D. discoideum* of different mating types, if approaching starvation under dark and humid conditions, may fuse to form a zygote giant cell. This diploid cell attracts nearby cells to form a precyst enclosed by a cellulose wall; this, in turn, matures into a dormant macrocyst, which germinates if conditions allow to release tens or hundreds of haploid progenies. (**C**) In this assay, chimeric constructs are defined as MatA-type if they allow cells to form macrocysts with a type II tester strain. (**D**) Schematic illustrating the roles of each *mat* gene in crosses between different mating types: Either *matA* or *matB* is required for mating compatibility with type III strains, whereas *matA* also confers mating with strains carrying *matC* (*24*). (**E** to **G**) Only chimeras with the C-terminal tail of MatA confer mating compatibility with type II cells, implying that this region is principally responsible for the different functions of MatA and MatB, at least within this context.

To ask whether the core DNA binding region or the tails of MatA and MatB determine their different functions, we produced a set of “chimeric” constructs, in each of which the divergent tail regions and cores of the two proteins were differently combined (Fig. 4A). These chimeric constructs were expressed in *mat* null *D. discoideum* cells, and the mating behavior of the resulting strains was studied to determine which chimeric constructs behaved similarly to MatA or MatB in vivo. To identify those with MatA-type activity, we used a macrocyst formation assay: Macrocysts are the walled dormant structures formed by dictyostelid zygotes only after fusion of cells of compatible mating types (Fig. 4B). Mixtures of chimera-expressing strains and type I, II, and III wild-type tester strains were incubated under dark and submerged conditions, and observation of macrocysts was taken to indicate mating compatibility between the two strains (Fig. 4C). As expected, because either MatC or MatS is required to specify mating compatibility with type I (Fig. 4D) (*24*), none of the chimera-expressing strains produced macrocysts with the type I tester strain (Fig. 4E), but they all successfully mated with the type III strain (Fig. 4G), demonstrating that, in this context, both MatA and MatB are functional even when their tails are exchanged. The distinguishing feature of *D. discoideum* cells expressing MatA alone, as opposed to those expressing only MatB, is their ability to mate with type II cells (*24*). Strikingly, we found that type II cells only produced macrocysts in crosses with cells expressing chimeric constructs containing the C-terminal region of MatA (Fig. 4F). The central DNA binding region of MatA had no effect in this assay in the absence of the MatA C terminus, suggesting that the homeodomain-like domains of MatA and MatB may recognize similar or identical DNA motifs. These data imply that differences between the C-terminal tails of MatA and MatB are critical in allowing cells expressing MatA, but not MatB, to mate with type II cells.

### Homeosis of life cycle stages in *mat* mutants

**Fig. 5:**
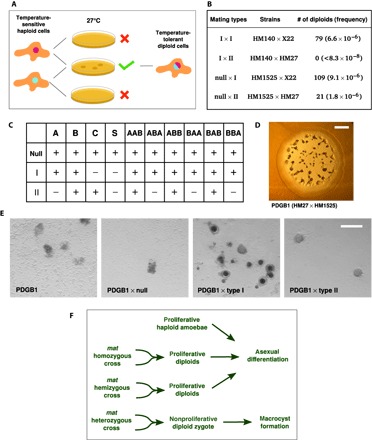
The mat locus controls the growth mode of *Dictyostelium* diploids. (**A**) Parasexual diploids were selected by mixing temperature-sensitive mutants, allowing low-frequency cell fusions at the permissive temperature, and then plating cells in cocultures with *Klebsiella* bacteria at the restrictive temperature. (**B**) In type I plus type II mixtures, no plaques of growing amoebae appeared, corroborating earlier findings (*33*); in contrast, a *matA* null strain in the same type I background readily produced plaques of temperature-tolerant diploids. (**C**) The contributions of other *mat* genes were tested by expressing green fluorescent protein (GFP)– or monomeric red fluorescent protein (mRFP)–tagged proteins in *matA* null strains, either using high-temperature selection for diploids in crosses with *mat* null, type I, and type II strains as above (for *matA*, *matB*, *matC*, and *matS*) or using double-drug selection (*52*). Growth and proliferation of diploids is represented by “+,” and absence of growth is represented by “−.” These results show that *mat* genes act to prevent growth and proliferation of sexual diploids, whether using bacteria or nutrient broth as food. (**D**) *mat* hemizygous diploids grow and develop asexually very similarly to haploids. Scale bar, 2 mm. (**E**) These hemizygous diploids form macrocysts when mixed with type I cells (NC4) but not with type II cells (V12M2), behaving in the same manner as type II haploid cells. Scale bar, 0.2 mm. (**F**) *Dictyostelium mat* mutants therefore display a form of homeosis in which diploid individuals behave as haploid (*35*): When type I cells from which *matA* has been deleted fuse with type II cells, the resultant diploid acts as a type II haploid and not a type I/type II zygote.

Macrocyst formation indicates that mating has been successful between two strains but does not distinguish functions of the *Dictyostelium* Mat proteins in specifying haploid function, for example, expression of cell recognition proteins, from roles in the diploid phase of macrocyst development. A locus closely linked to *mat* has previously been implicated in controlling the feeding or proliferative behavior of diploid cells in a phenomenon called “vegetative incompatibility.” When parasexual diploids are selected by growth under restrictive conditions, only crosses between strains of the same mating type are routinely successful; when strains of different mating types are crossed, the very rare diploids recovered were found to have become homozygous at *mat* (*33*). We confirmed that the *mat* locus itself is responsible for this phenomenon by deleting *matA* in a type I mutant that is unable to grow at high temperature and selecting for heat-tolerant parasexual diploids in crosses with other temperature-sensitive type I and type II strains (Fig. 5A). Unlike the parental type I strain, the *matA* null mutant produced parasexual diploids under restrictive growth in crosses with type II as well as type I strains (Fig. 5B). We also found that *matS* prevents the growth of diploids in crosses with both type I and type II cells, whereas *matC* prevents the growth of diploids in crosses with type I (Fig. 5C). Selections for the growth of parasexual diploids from crosses with cells expressing the chimeric MatA/B constructs confirmed the importance of the C termini of these proteins in their respective functions (Fig. 5C). Chimeras containing the C terminus of MatA prevent the growth of diploids in crosses with type II but not type I strains. These data indicate that interactions between Mat proteins, whether physical or genetic, induce zygotic functions after fusion of complementary cells and implicate the C terminus of MatB in enabling growth in the presence of MatC. The C terminus of MatA promotes zygote development in the same context, presumably leading to an exit from the mitotic cell cycle: Zygotes, as mentioned above, are not thought to proliferate mitotically, instead committing to meiosis before mitotic growth of haploid progeny can resume (*15*).

Diploids hemizygous at *mat*, like parasexual diploids resulting from crosses of the same mating type, grow on bacterial lawns and pass through the asexual cycle when food is depleted, forming fruiting bodies with stalks and spores, in a very similar manner as haploid cells (Fig. 5D). Spores formed by these parasexual diploids are markedly larger than those formed by haploids (fig. S12), in line with earlier findings (*34*), but appear otherwise identical to them. We were able to segregate haploid derivatives of these diploids by treating diploids with the microtubule-destabilizing agent thiabendazole; spores of these progenies are similar in size to their ultimate haploid parents (fig. S12). Strikingly, these hemizygous diploids containing the type II Mat proteins produce macrocysts when paired with type I cells (Fig. 5E); although we have not been able to confirm their karyotype, it is possible that these macrocysts are triploid. Collectively, these data indicate that *mat* hemizygous diploids behave identically to normal haploid *Dictyostelium* cells (Fig. 5F). This form of homeosis, in which organisms in one life cycle segment display the characteristics of another segment, has also been observed in other species (*35*) and often results from mutation of homeobox genes (*9*, *10*, *36*, *37*), suggesting that this function could be ancient and widely conserved despite radical differences in the life histories of the diverse lineages in which it has been observed.

## DISCUSSION

Because the polypeptide sequences of *Dictyostelium* MatA-like proteins displayed no obvious similarity to known protein families, their evolutionary origin was not clear. Our solution structures have revealed a clear relationship of their central helical fold with the homeodomain. At present, we cannot exclude the possibility that MatA and MatB evolved from another family of HTH transcription factors, but the parallels of their functions with those of homeodomain proteins that control sexual development in other organisms support the proposal that they descend from an ancestral amoebozoan homeobox gene. The C-terminal region of known MatA and MatB homologs is particularly variable, differing in length and sequence. This region is the most important in determining the distinct functions of MatA and MatB function in *D. discoideum*; the lack of homology in this region among dictyostelid MatA-like proteins (Fig. 2) suggests that it has diverged rapidly after insertion and deletion events rather than through incremental changes of single amino acid substitutions. Further genome sequences from dictyostelid species and their near relatives should clarify the nature of this variation, as well as shedding further light on the evolutionary processes that have spurred the divergence of their sequences.

**Fig. 6:**
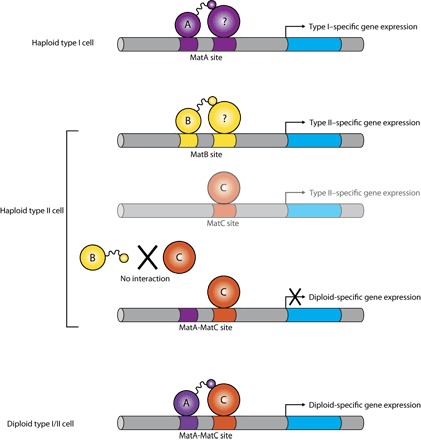
Hypothetical model for the regulation of mating-type determination and the haploid-diploid transition by the *Dictyostelium* Mat proteins. In the haploid type I cell, MatA binds to DNA in cooperation with (as yet unidentified) MatA-specific partner proteins via interactions involving its C-terminal tail, activating the expression of type I–specific genes. In the haploid type II cell, MatB interacts with its own distinctive partner proteins (again via its C-terminal tail) to bind DNA and activate expression of type II–specific genes. We hypothesize that MatC activates the expression of further type II–specific genes, but little is currently known about MatC to suggest how it functions. Following the fusion of type I with type II cells at the beginning of the sexual cycle, the diploid type I/type II cell now contains both MatA and MatC, and these proteins may interact via the C-terminal tail of MatA to activate the expression of diploid-specific genes. However, no such interaction occurs between MatC and the C-terminal tail of MatB in the haploid type II cell, and so, diploid-specific genes are not expressed. The Mat proteins may also cooperate to repress haploid-specific gene expression in diploid cells, whereas in haploid cells, they may be involved in repressing diploid-specific gene expression before cell fusion. In our model, we propose that MatS functions in a similar way to MatC in haploid type III cells to activate specific gene expression, and interactions between MatA/MatS and MatB/MatS pairs (similar to those between MatA/MatC in a type I/type II diploid cell) activate diploid-specific expression in type I/type III and type II/type III diploids, respectively.

Drawing upon similarities between our current knowledge of mating-type determination in *D. discoideum* and the mechanisms used by other species in which homeodomain proteins are involved (*5*, *8*), we suggest a hypothesis as to how MatA and MatB may function. In this model (summarized in Fig. 6), we propose that these proteins induce specific patterns of gene expression in haploid cells of each mating type that lead to fusion competence, likely in combination with cell type–independent partner proteins that respond to growth conditions and perhaps quorum-sensing factors. As in other systems, this process could involve up-regulating the expression of pheromones, pheromone receptors, or specific cell adhesion and cell fusion molecules (*25*), promoting the efficient fusion of compatible cells to form a diploid zygote that would contain a new combination of Mat proteins. These proteins could then act together in activating a set of diploid-specific genes, triggering the developmental program of macrocyst formation. Further investigations will be needed to identify the immediate downstream targets of these DNA binding proteins in haploid and diploid cells and to establish whether the relatively weak binding observed in vitro is a result purely of the noncognate sequences used in the experiments described above or is, in part, a property of these divergent proteins themselves. Homeodomains often bind to their cognate binding sequences with affinities in the nanomolar range or below (*38*, *39*), although another sex-determining protein, *S. cerevisiae* MATa1, binds as a monomer with a much lower affinity (*40*).

We previously proposed that mating type II arose after recombination of the *mat* locus in a cross between two preexisting mating types, the ancestors of types I and III (*24*). This implies that the ancestors of *matB* and *matC* were once competent to trigger zygote development but evolved to become incompatible with each other. This study suggests that an important part of this process involved changes to the C-terminal tail of MatB. Exchanging this region of MatB with the corresponding tail from MatA is sufficient to confer compatibility of haploid cells with type II partners, as well as promoting zygote development in diploids. Identification of binding partners, in haploid or diploid cells, will also aid further detailed structural analyses of the differences between the C termini of MatA and MatB. The parsimonious mechanistic explanation is that heterodimers are formed by an interaction of these C-terminal tails with MatC or MatS. Although MatA and MatB will almost certainly be coexpressed in type I/type II diploids, we have no reason to believe that a dimer between these two proteins has any important function, because MatB is dispensable for macrocyst formation in these crosses (*24*). By analogy with other instances where homeodomain proteins control sexual development, we would expect MatA and MatB to make dimeric interactions with another homeodomain-like protein. The molecular structures and functions of MatC and MatS remain unclear, although their localization in haploid cells is consistent with a role in transcriptional regulation (fig. S13). No homology with homeodomains is discernible in the sequences of MatC and MatS, but it remains possible that downstream targets of these proteins could include homeodomain proteins that interact directly with MatA and MatB.

Some other dictyostelid species have a greater number of mating types than *D. discoideum*. We have proposed that these extra mating types could have arisen by further multiplication of *matA*- and *matS*-like gametologs (*15*). Our model predicts that each species (defined as a breeding group) should have only one mating type containing a *matA*-like gene but no *matS*-like gene and one mating type with a *matS*-like gene but no *matA*-like gene; all additional mating types are expected to have both *matA*- and *matS*-like genes. However, given the form of sexual selection likely to exist in this inherently conflictual system, in which haploid cells are in competition to form a zygote that is programmed to consume the less competitive cells, it remains possible that the mechanism of sex determination could be fluid in these organisms, shifting as new advantageous variants arise in genes acting at other levels of the regulatory hierarchy.

Mutations in genes that control the developmental processes that determine haploid- and diploid-specific functions, and most critically the switch that triggers zygote differentiation in sexual organisms, can be homeotic, best illustrated in organisms that are multicellular in both haploid and diploid phases (*35*). Our findings in *Dictyostelium* add another example of such a mutation affecting a homeobox-like gene. This form of developmental control by homeodomain transcription factors is best understood in budding yeast (*5*, *36*, *41*). By selecting for diploids containing different *mat* genes, we have demonstrated that the *mat* locus controls the haploid-to-diploid transition in *Dictyostelium*. Deletion of *matA* from a type I strain causes an effectively dominant homeotic mutation: Diploids formed between this strain and a type II partner do not differentiate as zygotes as diploids heterozygous at *mat* do, most likely involving an exit from the mitotic cell cycle. Instead, these hemizygous diploids grow and proliferate mitotically in the same way as haploid cells (and homozygous diploids). Therefore, as in budding yeast, *mat* genes act in a codominant manner to induce *Dictyostelium* zygotic function.

The dual role of certain homeodomain proteins in controlling sex determination and zygote differentiation in basidiomycetes and saccharomycete yeasts is well understood. Accumulating evidence in land plants that related proteins have a similar critical function in establishing the haploid-to-diploid transition suggests that this function might have descended from the common ancestor of fungi and plants (*4*, *10*, *42*). Our evidence that homeodomain-like proteins in Amoebozoa share this function in governing sexual development adds further force to this proposal. Further investigation into the dictyostelid sexual cycle should clarify the depth of homology retained in this clade. Examination of a broader sample of eukaryotes will be essential to test the hypothesis that this function of homeodomain proteins is truly ancestral, enabling a deeper and more detailed theory of the evolution of sex. The archaeal origins of the machinery of sex (*2*) further suggest that homeodomain proteins may have first evolved, perhaps from genes similar to those encoding extant HTH-10 proteins, to govern programmed ploidy changes even before the emergence of eukaryotes.

## MATERIALS AND METHODS

### Protein purification

MatA and MatB constructs used for expression were based on pET28a containing the coding sequence for the *Bacillus stearothermophilus* dihydrolipoamide acetyltransferase lipoyl domain as an N-terminal expression tag. The lipoyl domain itself had an N-terminal His_6_-tag, and the vectors contained a tobacco etch virus (TEV) cleavage site between the tag and the *mat* sequences.

Expression of MatA and MatB proteins was performed in BL21(DE3) *Escherichia coli*. Cells were grown in either 2× TY for unlabeled samples or M9 minimal medium supplemented either with ^15^NH_4_Cl or with ^15^NH_4_Cl and [^13^C_6_]glucose (Sigma-Aldrich ISOTEC), depending on the desired labeling scheme, as the sole nitrogen or carbon source. Cells were grown to an optical density of approximately OD_600_ = 1 and cooled to 20°C, and protein expression was induced by addition of 0.5 mM isopropyl-β-d-thiogalactopyranoside. After further incubation at 20°C overnight, cells were harvested by centrifugation.

All purification steps were carried out at 4°C or on ice. Cell pellets were resuspended in lysis buffer containing 50 mM tris-HCl (pH 8), 750 mM NaCl, 10 % (w/v) sucrose, 1 mM phenylmethylsulfonyl fluoride, and protease inhibitor mix (Roche EDTA-free cOmplete Protease Inhibitor Cocktail; 1 tablet per 50 ml) and lysed by sonication, and the cleared lysate was incubated with nickel–nitrilotriacetic acid (Ni-NTA) agarose resin (Qiagen) for 90 min at 4°C. The resin was washed with 150 ml of wash buffer [50 mM tris-HCl (pH 8) and 750 mM NaCl], and the bound protein was eluted with a buffer containing 50 mM tris-HCl (pH 8), 750 mM NaCl, and 350 mM imidazole. Elution fractions were pooled, concentrated, and purified by gel filtration using a Superdex 75 column (GE Healthcare), equilibrated in 50 mM tris-HCl (pH 7.4) and 500 mM NaCl. Desired fractions were pooled and cleaved with the TEV protease overnight, Ni-NTA agarose was used to remove the fusion tag and protease, and the protein was purified to homogeneity using a Superdex 75 column as before. Fractions containing the required protein were pooled and dialyzed against 4 liters of dialysis buffer [25 mM phosphate (pH 6), 100 mM NaCl, and 50 μM EDTA] overnight at 4°C.

### Electrophoretic mobility shift assay

Polyacrylamide gels for EMSAs were cast following the standard protocols; the gels used were 6% (v/v) polyacrylamide (37.5:1 acrylamide/bisacrylamide) with 1× tris-borate buffer (90 mM of each) in the gel and running buffers. Gels were prerun at 55 V for 30 min at room temperature. DNA-protein samples were incubated in 10 mM tris-HCl (pH 7.4), 1 mM EDTA, and 20 mM NaCl for 30 min at room temperature before addition of 4× loading buffer [10 mM tris (pH 7.4), 1 mM EDTA, 50% glycerol, 0.04% bromophenol blue], and 5 μl of samples was loaded onto the gel. Gels were run at 80 V for 75 min and then visualized by staining with SYBR Gold DNA stain (Thermo Fisher Scientific). dsDNA fragments used in these experiments were prepared using DNA oligonucleotides produced by Integrated DNA Technologies. The following sequences were used: 5′-CCGAAACGTTTGGTGGCGCATGTAATTCATTTACACGCGCGAGGGCGTGCAAGATTC-3′ and 5′-GAATCTTGCACGCCCTCGCGCGTGTAAATGAATTACATGCGCCACCAAACGTTTCGG-3′.

### Alignments

Homologs of the Mat proteins from different *Dictyostelium* species were identified by blastp (*43*) using unpublished genomes available in public databases. One sequence, from *Dictyostelium intermedium*, appears to be misassembled, because the N-terminal region contains a long perfect repeat; this was removed. The *Dictyostelium giganteum matA/B* sequence was assembled from transcriptome sequence reads (Sequence Read Archive project SRP002432) using the Ray assembler (*44*). Multiple alignments were constructed using MAFFT (*45*) and displayed using CLUSTALX (*46*). The phylogenetic tree derives from a MAFFT alignment of selected homeodomain and HTH-10 proteins; the initial alignment was trimmed manually to include several residues in either side of the core HTH motif and then realigned. The tree was calculated using IQ-TREE (*47*, *48*), using the DG + G4 model; a consensus tree from 1000-replicate ultrafast bootstraps is shown. Dendroscope (*49*) was used to draw the tree. The structure-based alignment of MatA and MATα2 was produced using the FATCAT server (http://fatcat.burnham.org) using the FATCAT-pairwise algorithm (*27*). Input structures were the lowest energy structure of the final MatA ensemble from the AMBER calculations (see below) and the crystal structure of MATα2 from Protein Data Bank (PDB) 1APL (chain C).

### Growth and transformation of *Dictyostelium* cells

*Dictyostelium* cells were cultured axenically in HL5 broth (Formedium) or in association with *Klebsiella pneumoniae* on SM agar (Formedium). Cells were transformed by electroporation (*50*) and selected under axenic conditions. *matA* was deleted from the temperature-sensitive strain HM140, and the blasticidin resistance cassette was removed by Cre recombinase, using the constructs described previously (*24*). HM1559 is an alternative mating type–switched clone prepared alongside HM1528, a strain described earlier (*24*). Strains used in this study are listed in table S2. Fusions of Mat proteins with GFP or FusionRed for localization experiments were expressed using the pDM1005 vector or a derivative therefrom, with the GFP coding sequence replaced with that of FusionRed. Growing cells were imaged using a Zeiss laser scanning confocal microscope.

### Construction of chimeric MatA/MatB sequences

Identical boundaries for the N-terminal, central, and C-terminal regions were defined for both MatA and MatB based on their homology; residues 27 to 86 were defined as the central region because they are flanked by stretches of nonconserved residues. To produce the coding sequences of the MatA/MatB chimeras, fragments of the MatA and MatB DNA sequences were amplified by polymerase chain reaction (PCR) using KOD Hot Start DNA Polymerase (Novagen) as per the manufacturer’s instructions such that the PCR products contained overlapping regions and could subsequently be combined to use as a template in a further PCR. The constructs were first cloned into bacterial vectors and then subcloned into a *D. discoideum* expression vector based on pDM304 (*51*) containing a C-terminal GFP tag.

### Macrocyst formation assay

Heat-killed *K. pneumoniae* used in the macrocyst crosses were produced by growing up an overnight culture of the bacteria in LB medium, harvesting the cells by centrifugation, and washing three times in MSS buffer [5 mM MES (pH 6), 10 mM NaCl, 10 mM KCl, 10 mM CaCl_2_]. Cells were then heated at 60°C for 20 min before cooling and storing at −20°C. Cells to be crossed were grown separately in suspension with heat-killed *K. pneumoniae* (diluted to OD_600_ = 10) in MSS buffer with tetracycline (10 μg/ml) and streptomycin (200 μg/ml) overnight and protected from light. Those strains that had grown to a density of 10^6^ cells/ml were washed three times in fresh MSS before mixing. For crosses, 5 × 10^5^ cells of each strain were mixed with 50 μl of an OD_600_ = 10 suspension of heat-killed bacteria in 500 μl of MSS per well in 24-well plates and incubated in the dark at 22°C for 7 days. Macrocysts were visualized for counting using a light microscope.

### Parasexual crosses

To select parasexual diploids, haploid cells grown on SM agar plates in association with *K. pneumoniae* were washed free of bacteria in MSS buffer and 10^6^ cells of each strain were mixed in a total of 1 ml of MSS in 24-well tissue culture plates. These mixtures were shaken at 180 rpm at 22°C overnight to allow cell aggregates to form, promoting low-frequency fusion. Aggregates were broken up by repeated trituration, and then cells were cultured under selective conditions that permit diploid but not haploid cells to grow. For selection by temperature-resistant growth, haploid strains bearing mutations rendering them unable to grow at high temperature were mixed; in each mixture, a different gene is mutated so that, in diploids formed between them, each mutation is complemented by the wild-type gene. To select against temperature-sensitive haploids, cells were grown in association with *K. pneumoniae* on SM agar at 27°C for several days in a humid container. In double-drug selections (*52*), *matA* null cells transformed with *mat* gene (fused to GFP or mRFP) expression constructs bearing a G418 resistance cassette were crossed with tester haploid strains carrying a blasticidin resistance cassette. To select against drug-sensitive haploids, cells were plated in 10-cm tissue culture dishes in HL5 broth plus 10 μM blasticidin S and 20 μM G418. Because these experiments resulted in growth or absence of growth, we did not quantify the frequency of diploid formation in the double-drug selections; in each cross, at least 5 × 10^6^ cells were screened in multiple independent experiments. Spore lengths were measured using the program Fiji (*53*). Haploid segregants were obtained from parasexual diploids by treatment with thiabendazole (2 μg/ml) during growth on *K. pneumoniae* in SM/5 broth. Cells were cloned on SM agar after 4 days of thiabendazole treatment: Slower growing plaques were selected as potential haploids and then spore size, shape, and color were used to confirm ploidy and genotype. *mat* genotype was checked by PCR.

### Size exclusion chromatography–multiangle light scattering

Size exclusion chromatography (SEC)–MALS was carried out using a Wyatt Heleos II 18-angle light scattering instrument coupled to a Wyatt Optilab rEX online refractive index detector. Samples were exchanged into 20 mM phosphate (pH 7), 50 mM NaCl, and 2 mM EDTA and loaded onto a Superdex 75 10/300 GL column (GE Healthcare) running at 0.5 ml/min and preequilibrated in a sample buffer. A bovine serum albumin standard was run before the Mat samples to generate a calibration curve, and SEC-MALS and QELS (quasi-elastic light scattering) data were analyzed using the manufacturer’s ASTRA software.

### CD spectroscopy

CD spectra were measured using a Jasco J-810 spectrometer, scanning in the range of 260 to 190 nm at 20°C with samples of MatA or MatB in 50 mM phosphate (pH 6) and 100 mM NaCl.

### NMR spectroscopy

NMR samples of MatA comprised 0.3 to 0.6 mM ^15^N- or ^15^N,^13^C-labeled protein solutions in 50 mM ^2^H_11_ tris buffer (pH 7), 100 mM NaCl, 1 mM ^2^H_6_ dithiothreitol, and 50 μM EDTA in 95:5 H_2_O/^2^H_2_O; NMR samples of MatB comprised 0.3 to 1 mM ^15^N- or ^15^N,^13^C-labeled protein solutions in 25 mM phosphate buffer (pH 6), 100 mM NaCl, and 50 μM EDTA in 95:5 H_2_O/^2^H_2_O. NMR data were acquired using Bruker DMX600, DRX600, and Av-1 800 spectrometers, each equipped with a cryogenically cooled triple-resonance (^1^H/^15^N/^13^C) 5-mm probe. Experiments were conducted at 15°C (MatA) or 1°C (MatB), and ^1^H chemical shifts were calibrated using sodium 3,3,3-trimethylsilylpropionate as an external ^1^H reference; ^15^N and ^13^C chemical shifts were indirectly referenced to the ^1^H shifts using the ratio of gyromagnetic ratios (*54*). The following were acquired for MatA and MatB: two-dimensional (2D) data sets: [^15^N-^1^H] HSQC, [^13^C-^1^H] HSQC covering the full ^13^C spectral width, and constant-time [^13^C-^1^H] HSQC covering only the aliphatic ^13^C region; 3D data sets: HNCACB, CBCA(CO)NH, HNHAHB, HBHA(CO)NH, [^1^H-^13^C-^1^H] HCCH-TOCSY, [^1^H-^13^C-^1^H] HCCH-COSY, [^13^C-^13^C-^1^H] HCCH-TOCSY, ^15^N NOESY-HSQC (τ_m_ = 150 ms), and ^13^C NOESY-HSQC (τ_m_ = 150 ms); separate data sets were acquired for ^13^C aliphatic and aromatic spectral regions. All of the NOESY data sets used for structure calculations (see below) were acquired using pulse sequences modified to ensure equal RF heating in each case, for example, for ^13^C experiments, a period of ^15^N decoupling equal in length to the acquisition period was applied at the beginning of the interscan delay, and for ^15^N experiments, an equivalent period of ^13^C decoupling was similarly applied. All spectra were processed using the program TOPSPIN versions 3.1 and 3.2 (Bruker GmbH) and analyzed using the program CCPN analysis (*55*).

Resonance assignments for MatA were made exclusively using the scalar coupling–based backbone experiments listed above; the resulting assignment completeness for MatA as reported by the UNIO software was ^1^H (94.78%), ^13^C aliphatic (90.87%), ^13^C aromatic (78.95%), and ^15^N (81.08%). In the case of MatB, the quality of the data from scalar coupling–based backbone experiments was markedly lower, and additional use had to be made of NOE-based experiments for assignments in protein regions that suffered the most severe line broadening (these included particularly parts of the N-terminal tail); the final assignment completeness for MatB as reported by the UNIO software was ^1^H (82.64%), ^13^C aliphatic (83.82%), ^13^C aromatic (36.36%), and ^15^N (79.49%).

^15^N{^1^H} heteronuclear NOEs for the amide signals of MatA and MatB were determined using 250 and 120 μM solutions of ^15^N-labeled MatA and MatB, respectively, adjusted to 25 mM phosphate (pH 6), 100 mM NaCl, and 50 μM EDTA with 5% D_2_O (v/v), essentially as described by Skelton *et al*. (*56*) and using a saturation time of 7 s. All data were collected at 1°C on a Bruker DRX600 spectrometer equipped with a triple-resonance (^1^H/^15^N/^13^C) cryoprobe. The ^15^N{^1^H} heteronuclear NOE data were collected in an interleaved manner, and ^15^N{^1^H} heteronuclear NOE values were calculated from the ratio of peak intensities of pairs of spectra acquired with and without ^1^H saturation; the error bars shown in Fig. 1 (E and F) are SDs calculated according to Eq. 3 of Farrow *et al*. (*57*).

### Shift perturbation analysis

Backbone amide CSP analyses were performed for samples of ^15^N-labeled MatA upon addition of the various fragments of unlabeled dsDNA listed below. 2D ^15^N-^1^H HSQC spectra were acquired at 25°C for samples containing 80 μM DNA and 20 μM MatA in 25 mM phosphate (pH 6), 50 mM NaCl, and 50 μM EDTA with 5% (v/v) D_2_O. CSPs were calculated for interaction with the 29-bp oligonucleotide, using the assignments described above according to the observed peak distributions in the ^1^H and ^15^N dimensions and applying the formula Δδ = √((Δδ(^1^H))^2^ + (Δδ (^15^N)/6.8)^2^). In the case of the 58-bp DNA, additional spectra were acquired at DNA concentrations of 40, 80, and 120 μM (with MatA concentration of 20 μM throughout), and spectra were also acquired for MatB S71A (20 μM) in the presence and absence of the 29-bp DNA (80 μM) at 1°C.

DNA oligonucleotide sequences were as follows: 58 bp, 5′-GAATCTTGCACGCCCTCGCTCAAGCCTTCGTCACTGGTCCCGCCACCAAACGTTTCGG-3′ (forward) and 5′-CCGAAACGTTTGGTGGCGGGACCAGTGACGAAGGCTTGAGCGAGGGCGTGCAAGATTC-3′ (reverse); 29 bp, 5′-CCGAAACGTTTGGTGGCGGGACCAGTGAC-3′ (forward) and 5′-GTCACTGGTCCCGCCACCAAACGTTTCGG-3′ (reverse); 21 bp, 5′-CCGAAACGTTTGGTGGCGGGA-3′ (forward) and 5′-TCCCGCCACCAAACGTTTCGG-3′ (reverse); and 15 bp, 5′-CGTTTGGTGGCGGGA-3′ (forward) and 5′-TCCCGCCACCAAACG-3′ (reverse).

### Structure calculations

Initial structures of MatA and MatB were calculated using the program UNIO (*58*), for which the input comprises the respective protein sequences, the full resonance assignment, and the following processed 3D NOESY spectrum files: ^15^N NOESY-HSQC (τ_m_ = 150 ms), ^13^C aliphatic region NOESY-HSQC (τ_m_ = 150 ms), and ^13^C aromatic region NOESY-HSQC (τ_m_ = 150 ms).

Next, structures were calculated using XPLOR-NIH (*59*). As input, these calculations used the set of NOE restraints generated by the final (seventh) cycle of UNIO, reformatted for use in XPLOR-NIH. Because the XPLOR-NIH calculations used *r*^−6^ summation for all groups of equivalent protons and nonstereospecifically assigned prochiral groups, and because no stereoassignments were made (and the assignment-swapping protocol within XPLOR-NIH for deriving stereoassignments indirectly during the structure calculation itself was not applied), all constraints involving protons within these groups were converted to group constraints (by using wildcards such as HB*). All lower bounds were set to zero (*60*). Structures were calculated from polypeptide chains with randomized ϕ and ψ torsion angles using a two-stage simulated annealing protocol within the program XPLOR-NIH, essentially as described in Argentaro *et al*. (*61*).

In the case of MatA, the NOESY data were of sufficient quality that the NOE restraints derived from the UNIO calculations resulted in well-converged structures with good violation and Ramachandran statistics. In contrast, MatB S71A has markedly lower thermal stability than MatA, and its NOESY spectra consequently contained broader, less intense, and more heavily overlapped signals from the structured core, also resulting in a proportionately higher contribution from the *t*_1_ noise arising from sharp signals of the N- and C-terminal tails. As a result, the NOE restraints derived from UNIO for MatB S71A included a significant proportion that corresponded to misassigned spectral artifacts, leading to degradation of the structural resolution and statistics. These artifacts were largely eliminated during multiple rounds of analysis of successive XPLOR-NIH calculations, at each stage combining analysis of violations and Ramachandran statistics with manual verification of cross peaks in the spectra.

The structures calculated in XPLOR-NIH were finally subjected to a further stage of energy minimization using a full force field as implemented in the program AMBER 11 (*62*). Calculations comprised 100 steps of steepest descent and then 1900 steps of conjugate gradient minimization; the experimental distance restraints (after format conversion from XPLOR-NIH and applied in AMBER 11 using the default weight of 1) and implicit water-solvent representation using the generalized Born method (igb = 1) were used throughout.

The program CLUSTERPOSE (*63*) was used to calculate the mean root mean square deviation (RMSD) of ensembles to their mean structures, and structures were visualized using the program PYMOL (*64*). Electrostatic surfaces were calculated using the programs APBS (*65*) and pdb2pqr (*66*). Hydrophobic surfaces (fig. S2, E and F) were computed using the hydrophobicity scale of Kyte and Doolittle (*67*). Ensembles were superposed using the coordinates of their respective average structures; the average structures themselves are not shown.

## Supplementary Material

http://advances.sciencemag.org/cgi/content/full/3/9/e1602937/DC1

## SUPPLEMENTARY MATERIALS

Supplementary material for this article is available at http://advances.sciencemag.org/cgi/content/full/3/9/e1602937/DC1 table S1. Structural statistics. table S2. Strains used in this study. fig. S1. SEC-MALS and CD data for MatA and MatB. fig. S2. RMSD and AMBER energy profiles for the 50 calculated structures of MatA and MatB. fig. S3. Views of the core homeodomain-like region of MatA. fig. S4. 2D ^15^N-^1^H HSQC spectra of MatA and MatB. fig. S5. The MatB S71A mutant. fig. S6. Secondary chemical shift data for MatA and MatB. fig. S7. Distant homology shared between *Dictyostelium* Mat proteins, homeodomains, and archaeal HTH domains. fig. S8. Provisional phylogenetic placement *Dictyostelium* Mat proteins, homeodomains, and archaeal HTH domains. fig. S9. Model of a potential DNA binding mode of MatA. fig. S10. The DNA binding activity of MatB S71A. fig. S11. CSPs for MatA as a function of added 58-bp DNA concentration. fig. S12. Spore size of haploid and parasexual diploid strains. fig. S13. Localization of Mat proteins tagged with fluorescent proteins. References (*68*–*76*)
