# Supplementary material for: Homeodomain-like DNA binding proteins control the haploid-to-diploid transition in Dictyostelium
Source: Sci Adv. 2017 Sep 1;3(9):e1602937. doi: 10.1126/sciadv.1602937 (PMC5580921; doi:10.1126/sciadv.1602937)
Supplement: http://advances.sciencemag.org/cgi/content/full/3/9/e1602937/DC1 [file supp_3_9_e1602937__index.html]

Science Advances | Science Advances

## Supplementary Materials

**This PDF file includes:**

- table S1. Structural statistics.
- table S2. Strains used in this study.
- fig. S1. SEC-MALS and CD data for MatA and MatB.
- fig. S2. RMSD and AMBER energy profiles for the 50 calculated structures of MatA and MatB.
- fig. S3. Views of the core homeodomain-like region of MatA.
- fig. S4. 2D 15N-1H HSQC spectra of MatA and MatB.
- fig. S5. The MatB S71A mutant.
- fig. S6. Secondary chemical shift data for MatA and MatB.
- fig. S7. Distant homology shared between *Dictyostelium* Mat proteins, homeodomains, and archaeal HTH domains.
- fig. S8. Provisional phylogenetic placement *Dictyostelium* Mat proteins, homeodomains, and archaeal HTH domains.
- fig. S9. Model of a potential DNA binding mode of MatA.
- fig. S10. The DNA binding activity of MatB S71A.
- fig. S11. CSPs for MatA as a function of added 58-bp DNA concentration.
- fig. S12. Spore size of haploid and parasexual diploid strains.
- fig. S13. Localization of Mat proteins tagged with fluorescent proteins.
- References (*68–76*)

Download PDF

**Files in this Data Supplement:**

- Adobe PDF - 1602937\_SM.pdf
