# Supplementary material for: Homeodomain-like DNA binding proteins control the haploid-to-diploid transition in Dictyostelium
Source: Sci Adv. 2017 Sep 1;3(9):e1602937. doi: 10.1126/sciadv.1602937 (PMC5580921; doi:10.1126/sciadv.1602937)
Supplement: http://advances.sciencemag.org/cgi/content/full/3/9/e1602937/DC1 [file 1602937_SM.pdf]

Science Advances

AAAS

advances.sciencemag.org/cgi/content/full/3/9/e1602937/DC1

Supplementary Materials for

Homeodomain-like DNA binding proteins control the haploid-to-diploid transition in *Dictyostelium*

Katy Hedgethorne, Sebastian Eustermann, Ji-Chun Yang, Tom E. H. Ogden, David Neuhaus, Gareth Bloomfield

Published 1 September 2017, *Sci. Adv.* **3**, e1602937 (2017)
DOI: 10.1126/sciadv.1602937

This PDF file includes:

- table S1. Structural statistics.
- table S2. Strains used in this study.
- fig. S1. SEC-MALS and CD data for MatA and MatB.
- fig. S2. RMSD and AMBER energy profiles for the 50 calculated structures of MatA and MatB.
- fig. S3. Views of the core homeodomain-like region of MatA.
- fig. S4. 2D ^{15}N-^{1}H HSQC spectra of MatA and MatB.
- fig. S5. The MatB S71A mutant.
- fig. S6. Secondary chemical shift data for MatA and MatB.
- fig. S7. Distant homology shared between *Dictyostelium* Mat proteins, homeodomains, and archaeal HTH domains.
- fig. S8. Provisional phylogenetic placement *Dictyostelium* Mat proteins, homeodomains, and archaeal HTH domains.
- fig. S9. Model of a potential DNA binding mode of MatA.
- fig. S10. The DNA binding activity of MatB S71A.
- fig. S11. CSPs for MatA as a function of added 58-bp DNA concentration.
- fig. S12. Spore size of haploid and parasexual diploid strains.
- fig. S13. Localization of Mat proteins tagged with fluorescent proteins.
- References (*68–76*)

Supplementary Materials

table S1. Structure statistics. Structural statistics for the deposited ensembles of structures for MatA and MatB.

**Structural restraints**	**MatA**	**MatB**
NOE-derived distance restraints		
Intraresidue	346	317
Sequential	556	680
Medium (2≤|i-j|≤4)	475	548
Long (|i-j|>4)	250	166
Total	1627	1711
**Statistics for accepted structures**	**MatA**	**MatB**
Number of accepted structures	30	30
Mean AMBER energy terms (kcal mol $^{-1}$ ± S.D.)		
E(total)	-3589.0 ± 15.6	-4184.4 ± 17.0
E(van der Waals)	-616.4 ± 10.0	-618.2 ± 9.9
E(distance restraints)	29.1 ± 3.0	19.4 ± 2.4
Distance restraint viols. > 0.2Å (average number per structure)	5.3 ± 2.1	1.7 ± 1.4
RMS deviations from the ideal geometry used within AMBER		
Bond lengths	0.0101 Å	0.0105 Å
Bond angles	2.04°	2.05°
**Ramachandran statistics**	**MatA Res. 35-79**	**MatB Res. 35-79**
Most favoured	91.2%	80.6%
Additionally allowed	8.1%	15.3%
Generously allowed	0.7%	4.1%
Disallowed	0.0%	0.0%
**Average atomic RMS deviations from the average structure (± S.D.)**	**MatA Res. 35-79**	**MatB Res. 35-79**
(N, Cα, C' atoms)	0.31 ± 0.06 Å	0.36 ± 0.09 Å
(All heavy atoms)	0.67 ± 0.06 Å	0.76 ± 0.10 Å

table S2. Strains used in this study. Names of strains, names of parental strains (if any), mating types, and genotypes are given. Mating type 'IIb' and 'IIc' are partial type-II phenotypes, in that type IIb will mate with type III cells only and type IIc will mate with type I cells only. The PDGB diploids listed here are hemizygous null/*matBCD*, and so phenotypically type II. The source of strains are numbered according to the citation in the reference list except for those strains originating in this study (A); and (B) an unpublished strain generated by Peggy Paschke.

Strain	Parent	Mating type	Genotype	Source
AX2	AX1 (NC4 derived)	I	*axeA2, axeB2, axeC2, matA(1)*	(*68*)
HM140	NP2 (NC4 derived)	I	*axeA1, axeB1, axeC1, matA(1) tsgA1,cyc-905*	(*69*)
HM27	DM16 (HM3 x HM30; V12M2-derived)	II	*tsg-901* and/or *tsg-903, whi-900, cyc-900, matBCD(2)*	(*70*)
HM1524	AX2	null	*axeA2, axeB2, axeC2, matA(GB1), bsR*	(*24*)
HM1525	HM140	null	*axeA1, axeB1, axeC1, matA(GB1), tsgA1, cyc-905, bsR*	A
HM1526	HM1524	null	*axeA2, axeB2, axeC2, matA(GB1)*	(*24*)
HM1557	HM1525	null	*axeA1, axeB1, axeC1, matA(GB1), tsgA1, cyc-905*	A
HM1559	HM1526	II	*axeA2, axeB2, axeC2, matBCD(GB1), bsR*	A
HM1875	AX2	I	*axeA2, axeB2, axeC2, matA(1), LifeAct-mRFP, bsR*	B
HM2935	HM1557	IIc	*axeA1, axeB1, axeC1, matA(GB1), tsgA1, cyc-905, mRFP-matC, neoR*	A
HM2955	HM1557	IIb	*axeA1, axeB1, axeC1, matA(GB1), tsgA1, cyc-905, matB-GFP, neoR*	A
NC4(S)	wild isolate	I	*matA(1)*	(*71*)
PDGB1	HM27 x HM1525	II	(HM27 x HM1525 diploid)	A
PDGB4	HM27 x HM2955	II	(HM27 x HM2955 diploid)	A
PDGB5	HM27 x HM2935	II	(HM27 x HM2935 diploid)	A
PXGB21	PDGB1	null	*matA(GB1), whi+*	A
PXGB22	PDGB1	null	*matBCD, whi-*	A
PXGB23	PDGB1	null	*matBCD, whi-*	A
V12M2	V12	II	*matBCD(2)*	(*72*)
WS2162	wild isolate	III	*matST(1)*	(*73*)
X22	DP4 (M28 x NP14; NC4 derived)	I	*whiA1,tsgD12,tsgE13,acrA1, sprA1*	(*74*)

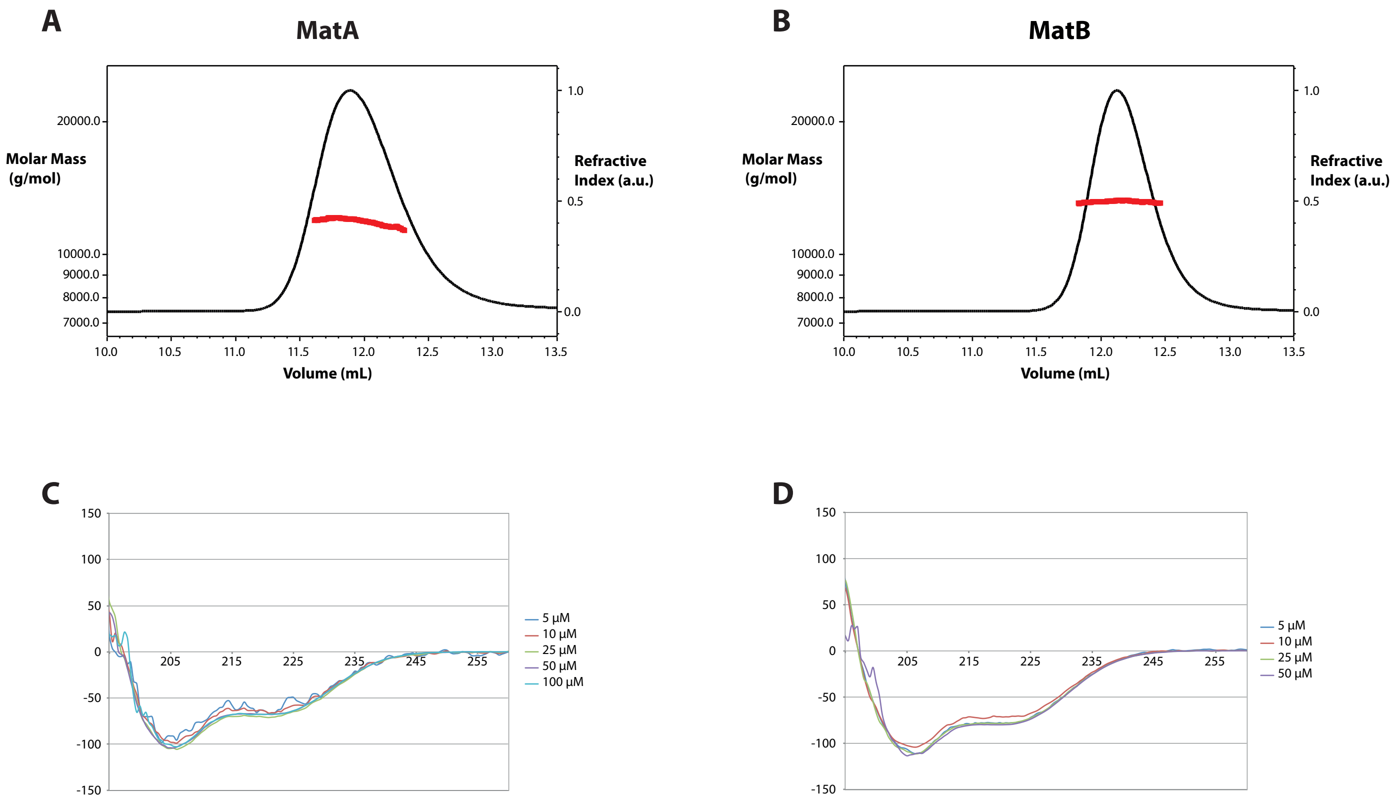

fig. S1. SEC-MALS and CD data for MatA and MatB. SEC-MALS experiments with both (**A**) MatA and (**B**) MatB clearly show them each to be monodisperse and monomeric in solution [MatA molecular mass 11.9 ± 0.1 kDa (theoretical 12.5), MatB molecular mass 13.2 ± 0.1 kDa (theoretical 12.6); molecular masses were calculated as averages across the region indicated and data were recorded in 20mM phosphate, 50mM NaCl, 2mM EDTA, pH7]. CD spectra for (**C**) MatA and (**D**) MatB show that in both cases the overall structure is independent of protein concentration over a wide range (spectra recorded in 50mM phosphate, 100mM NaCl, pH6; in each case, intensities have been multiplied by a normalization factor to aid comparison).

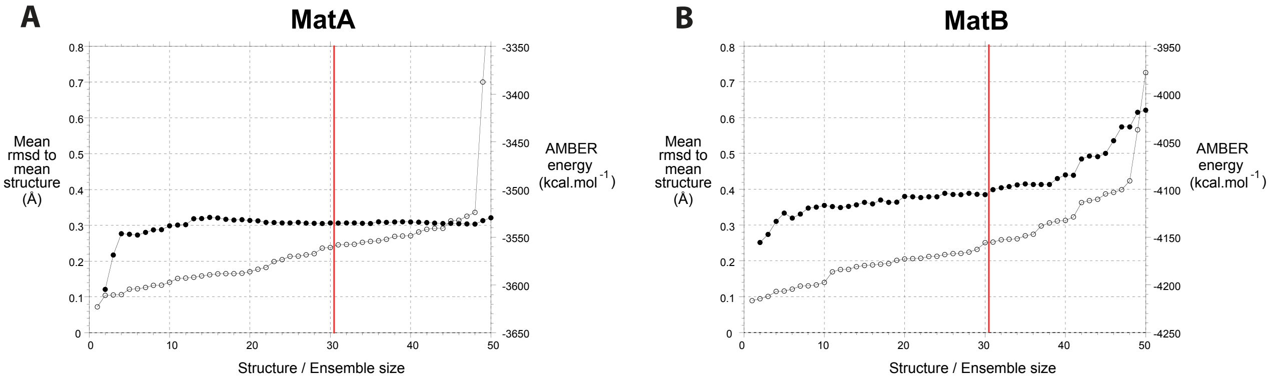

fig. S2. RMSD and AMBER energy profiles for the 50 calculated structures of MatA and MatB. Rmsd values (filled circles) are independently calculated for each ensemble size using the program CLUSTERPOSE (*63*), adding successive structures in order of increasing AMBER energy; open circles represent the AMBER energies of each structure. In each case, only the 30 structures to the left of the vertical red line were included in the deposited ensemble and when calculating the structural statistics.

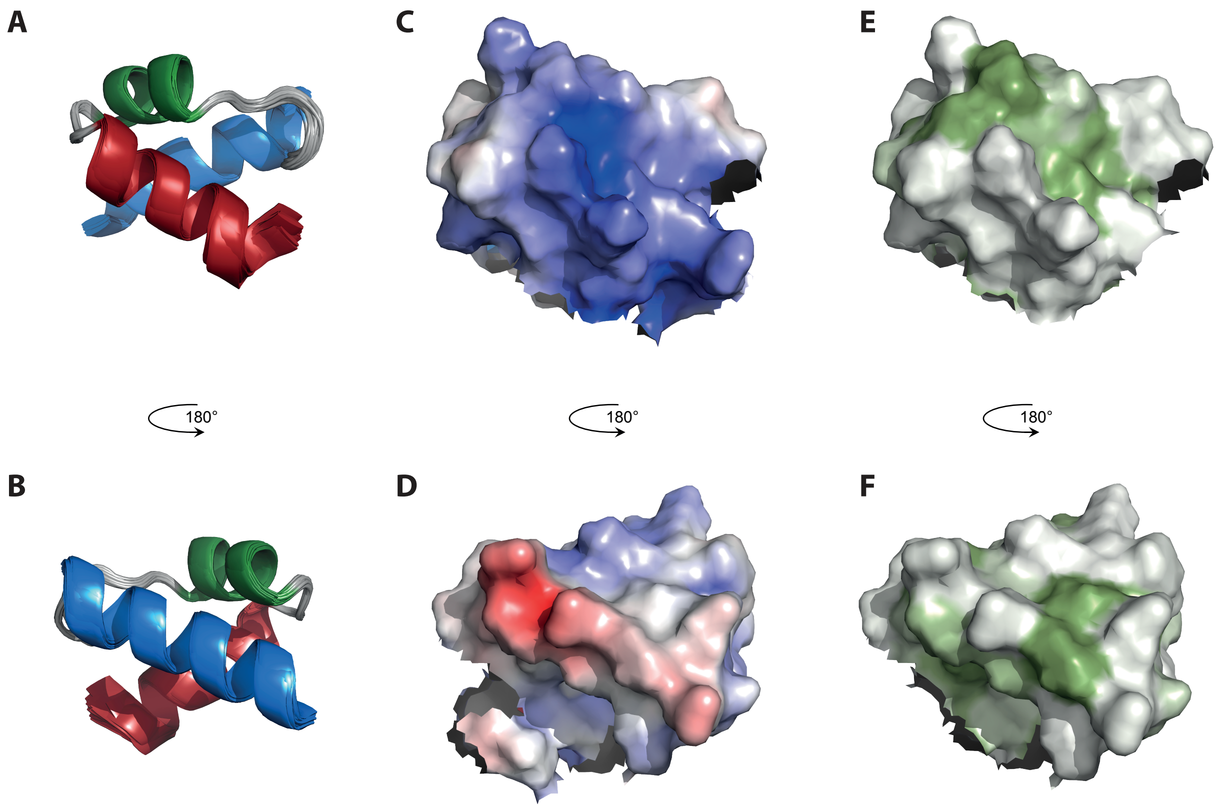

fig. S3. Views of the core homeodomain-like region of MatA. Showing the backbone cartoon representation (**A** and **B**), and the electrostatic (**C** and **D**), and hydrophobic (**E** and **F**) surfaces (panels A, C and D also appear in Fig. 1, and are repeated here to facilitate comparison). The disordered tails have been omitted from these views; relationships between the orientations of different structural views are indicated on the figure, and relative scalings of the views were chosen for clarity.

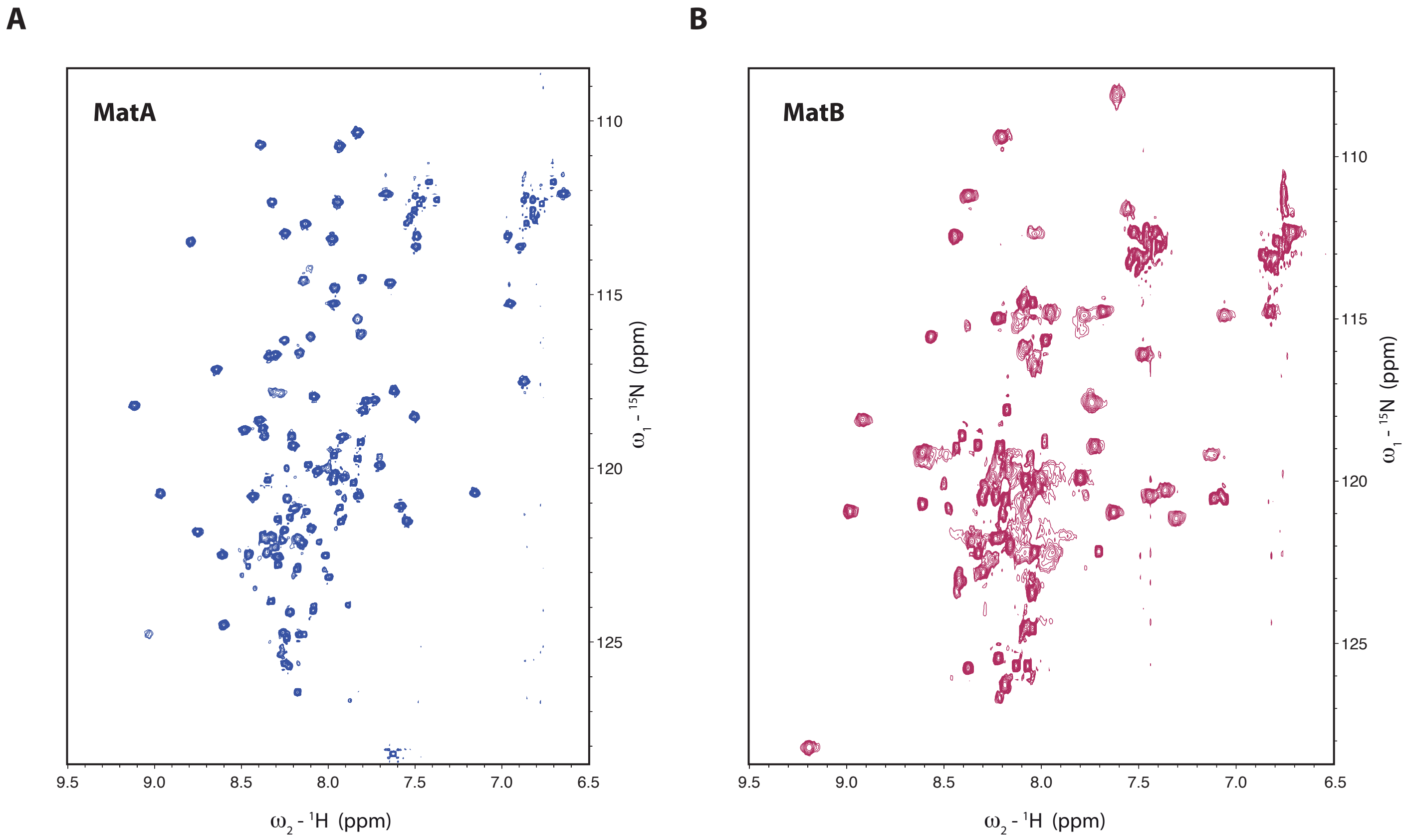

fig. S4. 2D ^{15}N-^{1}H HSQC spectra of MatA and MatB. The spectrum of MatB suffers from significant line broadening compared to that of MatA.

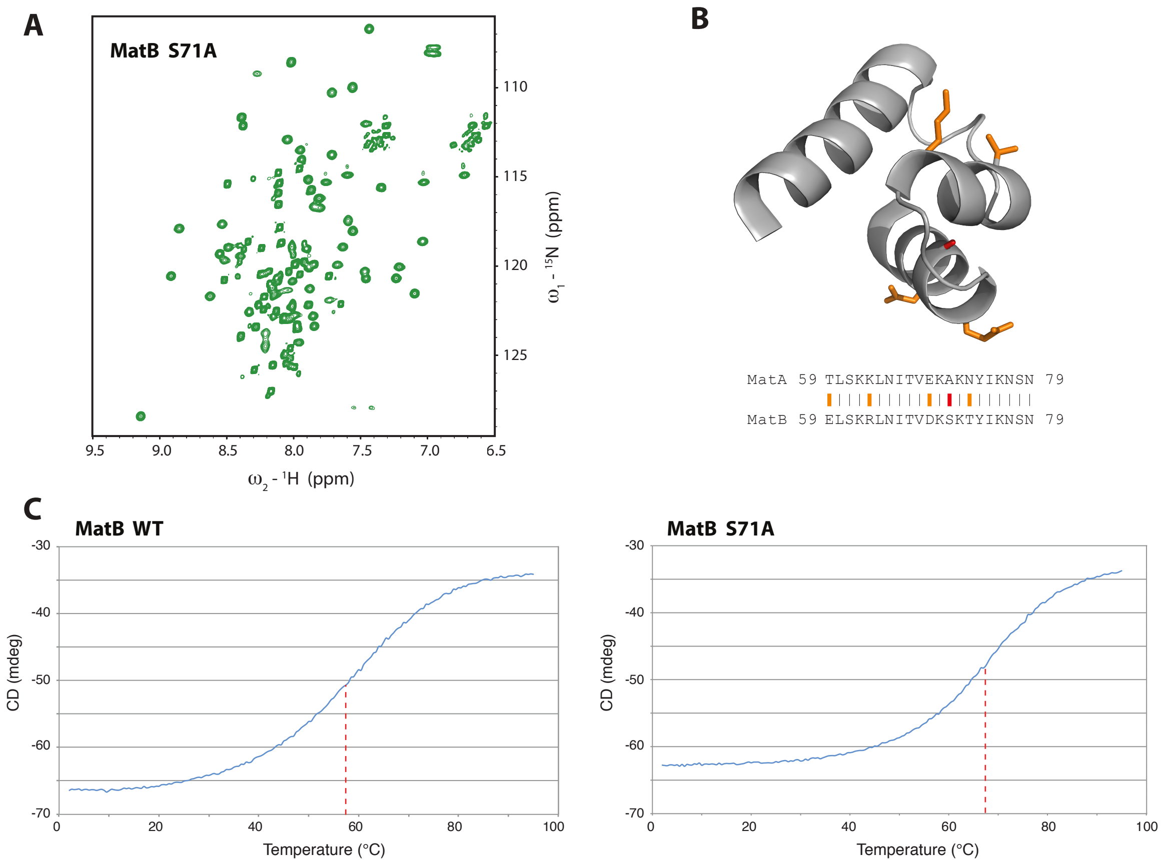

fig. S5. The MatB S71A mutant. The single point mutation S71A improved the quality of the NMR data such that an essentially complete resonance assignment could be obtained for this mutant. (**A**) The 2D [^{15}N-^{1}H] HSQC spectrum of S71A MatB was recorded at the reduced temperature of 1 ˚C and shows a considerable improvement, in terms of linewidth and peak resolution, compared to the wild type protein. (**B**) Inspection of the MatA structure suggests that the S71 side-chain of MatB is likely to be buried at the interface between helices 2 and 3, suggesting that the larger, more polar serine side-chain at this position in MatB causes destabilisation of the structure; differences between the MatA and MatB sequences in this region are indicated, and the corresponding sidechains are highlighted on the MatA structure. (**C**) Assessing the thermal stability of MatB and MatB S71A. Circular dichroism was followed at 222 nm as the samples were heated from 2-95 ˚C, and T_m values, indicated by the dashed red lines, correspond to the midpoint of the transition between the folded and unfolded states. These were calculated by fitting the data to a sigmoidal curve. The T_m value of wild-type MatB is 58 ˚C while that of MatB S71A is 68 ˚C; this suggests that it may indeed be an increase in the thermal stability of the mutant, relative to wild-type MatB, that results in the improvements seen in the NMR data recorded for this protein.

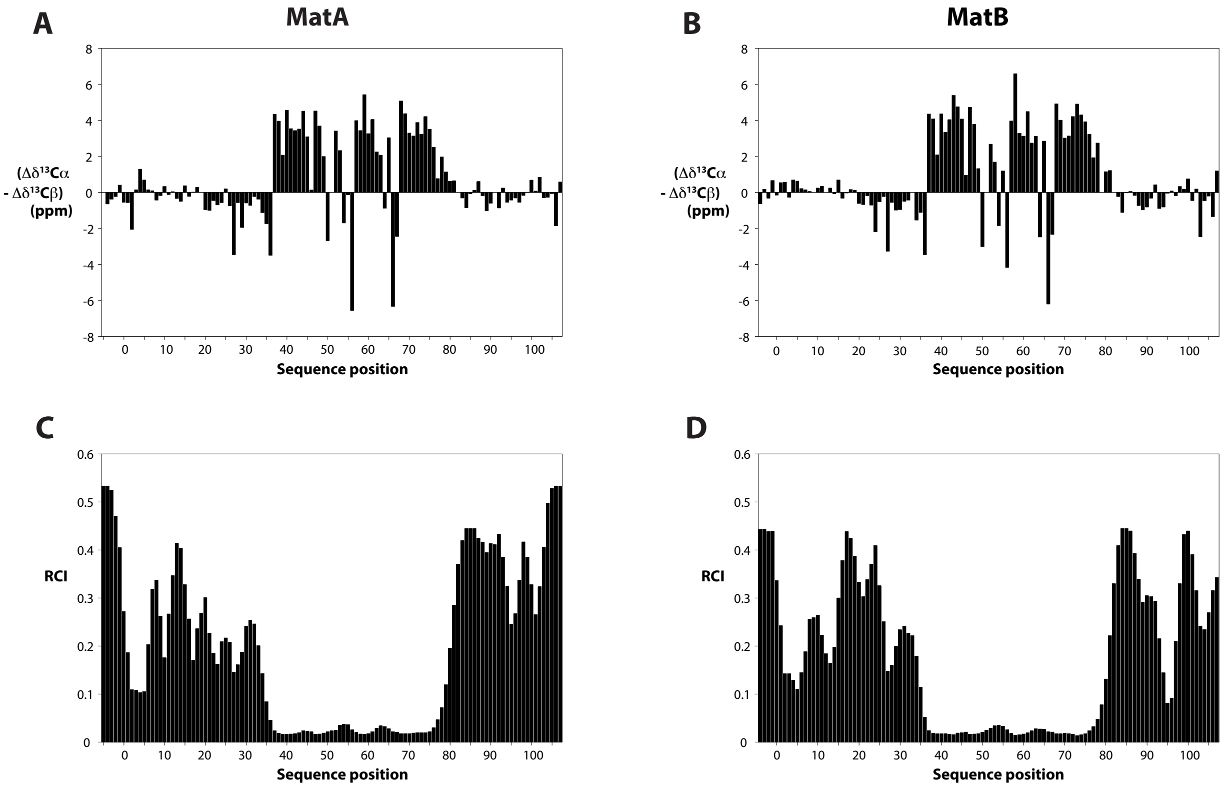

fig. S6. Secondary chemical shift data for MatA and MatB. Panels (**A**) and (**B**) show values of ($\Delta\delta^{13}C\alpha$ - $\Delta\delta^{13}C\beta$), where $\Delta\delta^{13}C\alpha$ is the difference between the experimentally measured value of $\Delta\delta^{13}C\alpha$ for a given residue and the corresponding random coil value, while $\Delta\delta^{13}C\beta$ is the corresponding quantity for Cβ (values were calculated using python scripts within the program CCPN analysis (*55*)). These plots show very similar patterns for MatA and for MatB. In each case, the N- and C-terminal tails show mainly small values suggesting they lack persistent structure, though for both MatA and MatB the region of residues approx. 20-40 shows values consistent with at least partial order. The patterns for the folded regions of MatA and MatB are also very similar between the proteins; in each case the three helices of the folded region correspond to regions with consistently large positive values of ($\Delta\delta^{13}C\alpha$ - $\Delta\delta^{13}C\beta$), while the turn regions also show similar patterns between MatA and MatB. Panels (**C**) and (**D**) show plots of the Random Coil Index (RCI) (*26*); for both MatA and MatB, the N- and C-terminal tails show high, though non-uniform, RCI values, consistent with our interpretation that these regions are largely unfolded but have some elements of residual structure, while the folded regions show uniformly low values. (N.B. all four panels include data for the 6-residue cloning artifact (GSHMAS) at the N-terminus, numbered -5 to 0).

```
A0A098_CHLRE RPKVGKLPPAATQLLKGWWDD--NFVWPYPSEEDKKQLGEAAALNNTQINNWFINQRKRHW
A9T288_PHYPA KRRAGKLPEGTTTVLKAWWQA--HSKWPYPTEDEKERLIQETGLELKQVNNWFINQRKRNW
A9SGQ5_PHYPA KRRAGKLPEGTTTVLKAWWQA--HSKWPYPTEDEKEQLIQETGLELKQVNNWFINQRKRNW
HBX9_DICDI   RKKRGKLPGEATSILKKWLFE--HNMHPYPTEEEKVALANSTFLSFNQINNWFTNARRRIL
HBX4_DICDI   PKKGAKLSKESKDILENWIKN--HIAHPYPTNDEKEQLQRQTGLTPNQISNWFINTRRRKV
HBX12_DICDI  KKNRRTLNDQYKSFISDYFKN--HSDHPYPNEDEKIIISALIDLSKYQRNNWFSNKRSREK
HBX3_DICDI   FKSRRILSEQQETNMNLWFDA--HVNNPYPEEDEKVILGAVNNLSKSQIDNWFGNKRMRDK
MTAL2_YEAST  PYRGHRFTKENVRILESWFAK--NIENPYLDTKGLENLMKNTSLSRIQIKNWVSNRRRKEK
MTAL2_KLUDE  PYRGHRFTKENVHTLEAWYSN--HIDNPYLDPKSLQSLAQKTNLSKIQIKNWVSNRRRKQK
MTAL2_PICAN  EKRSKRFPKTAQMELENWYTE--NEDNPYLSKRDLQQLVHKTGLCAPQVRNWVSNRRRKER
MTAL2_CANAL  KIKSRRLTKKQLLVLEGWFQK--HKNHPYSQKDQTNLLIKSTKLSKSQVQNWISNRRRKEK
HBX2_DICDI   KKRRTRLKKEQADILKTFFDN----DDYPTKDDKETLANRLGMSYCAVTTWFSNKRQEKK
WARA_DICDI   KKKRKRTSPDQLKLLEKIFMA----HQHPNLNLRSQLAVELHMTARSVQIWFQNRRAKAR
MATA1_YEASX  PKGKSSISPQARAFLEQVFRR----KQSLNSKEKEEVAKKCGITPLQVRVWFINKRMRSK
MATA1_PICAN  KKKRRHIPESSKELLEKAFKV----KRFPNSKERERIARECGISPLQVRVWFTNKRARSK
MATA1_KLUDE  HKRGCNIDKKTKDMLNKVYEQ----KQYLTKEEREFVAKKCNLTPLQVRVWFANKRIRNK
HBX6_DICDI   SGQRSLKTKEHKEILEALYRV----TLYPTSEETKTISQILGMTFGQVKSSFRHRREKLS
ANTP_DROME   KRGRQTYTRYQTLELEKEFHF----NRYLTRRRRIEIAHALCLTERQIKIWFQNRRMKWK
D8PSE1_SCHCM AQPVNLLKRARRPLLDRYFDL----NAYPSVTDKKALAAHEGATYRQIHVWFQNRRAKAR
CF1A_DROME   RKKRTSIEVSVKGALEQHFHK----QPKPSAQEITSLADSLQLEKEVVRVWFCNRRQKEK
HBX5_DICDI   SRRKNRFTDFQIKRMNDCFENLDKNNNGKFTSEEICQIATELGLTDQQVRVFFQNKRARSR
HBX13_DICDI  KRMRKTTRPDEKIYLEIYYQHF-YENNGKHSKDELITLSNNLNWKVNRIQRWLDNRRTKDK
HBX7_DICDI   NIRNIRSSGISTKKLEDFFSI----NQYPNKNEIKDFANYYQCDETKIKNWFKGKRDRLK
Dd_MatA      KPKLEELSEQQKIILAEYIAE--VGLQNI----TAITLSKKLNITVEKAKNYIKNSNRLGR
Dc_MatA      KPKARELSEEQKIILAEYITE--VGLHNI----TAITLSKKLDITLEKAQNYIKNN-RLSR
Dd_MatB      TQKTGELSEEQKKIVADYISE--VGLNNL----NATELSKRLNITVDKSKTYIKNSNRMGR
Di_MatAB     KPKKDELTKEQKIILAEYIQE--DAINSI----RAIDLAKRLNITVEKARSYLKNSKRSNR
Df_MatB      GPKTSELSKEQKIMVIDRILE--VGLDNI----TALDLSEKLNIPLKKAETYIKNSKRMER
Dg_MatAB     KPKANELTDEQKKIVVNFILE--VGAINI-TTQHSKQLSEKLDIPVDKAHHYLRNSLRSDR
E6P9F4_9ARCH FNLRAVLTPKQQQVLYTAFMM---GYFSPSRETSLSEIATRLGLSKSTVSRHLRTAMRKLA
Q4J721_SULAC MMILSSLTPTERQILYTAYKM---GFFDYPKKTKLEELAKMYGVTKVALDRHIRNAIRKVL
Q4J6W2_SULAC EIDESELTDRQLEILRLAYKS---GYFDVDRKISMKELANKLGIKASTLEEILRRALKKAV
E1QSW8_VULDI QLPMPSPTERQLEVLLLAYKM---GYFD--REVNLKELAKQLGLSISTVSELLRKTLKKVV
B1L500_KORCO VKRGRMLTERQEEVLLTAVRM---GYFDFPRRIRTRELADMLGMSQASLTEILRRAVKKLV
Q4JBG3_SULAC AKPSSIITGRQEQILKIALEL---GYFDFPRRIRLNELSKKLNISTSTLAEIIRRAERNII
E6N6W5_9ARCH VKPNGGLTSRQELIIKAALEL---GFFDYPKKIHVKELAQLFGITPATLTETMRKAMKRIV
G0EGP6_PYRF1 DEYDYMLTEKQERILIEAYLR---GYFSFPRKISMKDLAKELGMSVSSLAELLRKAEAKVV
E6P9G1_9ARCH IRAKHFLTPRQEQVLLHSYLN---GYFDNPRPIPLSKLAKDLGITPPSYLELLRKALKKVV
A1S0N6_THEPD TDLLSKLTPLQRKILSKAIIK---GYFDWPRKYSLSDLSQELGISKATLAEHIRRSESKIL
E1QSY1_VULDI SRLDVFLNSSEYKVLRHAFER---GFFNIPRSISMDELSKELGLSKSTIDRYLRSSLNKIL
Q4J6J7_SULAC DMFFPYLSPSQVRVLKAAFEY---GYLDYPREANADILAEKLNISKVTFLYHLRSAEKKLV
```

fig. S7. Distant homology shared between *Dictyostelium* Mat proteins, homeodomains, and archaeal HTH domains. The HTH regions of MatA/B-related proteins from dictyostelids were aligned with those from selected homeodomain, TALE homeodomain, and HTH-10 family proteins. One homeodomain from a Pou family transcription factor (CF1A_DROME) was also incorporated. Several *D. discoideum* homeodomain sequences (containing 'DICDI' in their identifiers) are included to emphasise the divergence of the Mat proteins from 'conventional' homeodomains, which are conserved across eukaryotes. Archaeal HTH-10 proteins are likely near-relatives of homeodomain proteins, although the precise evolutionary origin of the homeodomain remains unclear. A pattern of hydrophobic residues is conserved across the alignment, with the main structural difference being the length of the loop between helices 1 and 2. Positively charged residues are often present, without being strictly conserved, in the proximal sequences lying N-terminal to helix 1 and C-terminal to helix 3 in the Mat proteins and both homeodomain families, but typically only in the C-terminal part of HTH-10 proteins. Each sequence is identified by its UniProt entry name, except for the *Dictyostelium* Mat proteins, where 'Dd' is *Dictyostelium discoideum*, 'Dc' is *D. citrinum*, 'Df' is *D. firmibasis*, 'Dg' is *D. giganteum*, and 'Di' is *D. intermedium*.

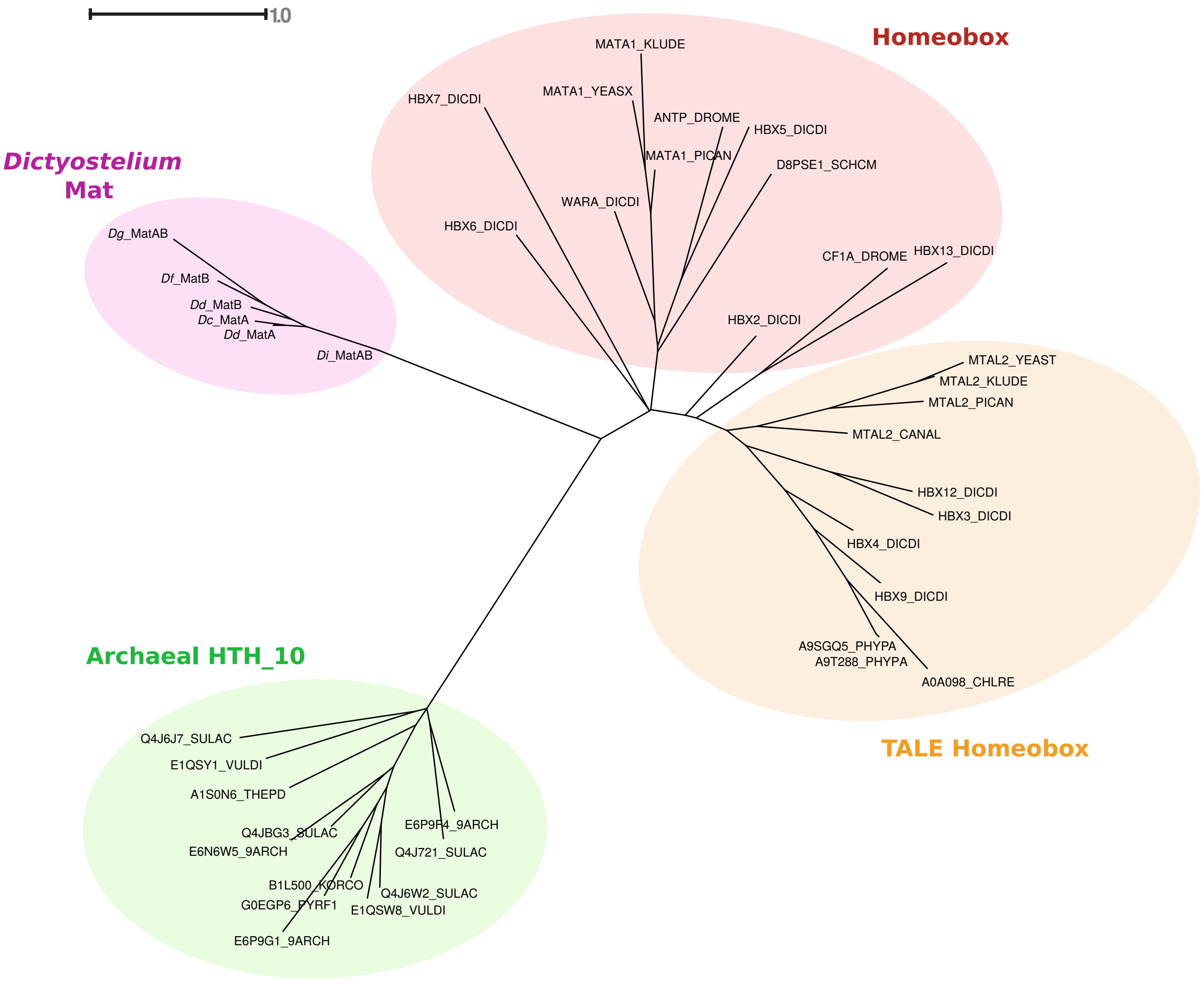

fig. S8. Provisional phylogenetic placement *Dictyostelium* Mat proteins, homeodomains, and archaeal HTH domains. A phylogram was constructed using the multiple sequence alignment in fig. S7 to provide a preliminary assessment of the phylogenetic placement of *Dictyostelium* Mat proteins. The same UniProt identifiers are used. The branch leading to the Mat proteins is long, even with the inclusion of 'conventional' *Dictyostelium* homeodomain sequences, making its placement difficult to determine. Based on functional similarities with known homeodomain proteins regulating sexual development, as well as the sequence and structural data presented in this study, we expect that their true position will be found to be nested within the tree of homeodomains (broadly speaking), having diverged since the origin of dictyostelia (or perhaps earlier in Amoebozoan evolution), but the data available to us now do not exclude the possibility that they arose from a horizontally-acquired bacterial or archaeal sequence. The identification of further Mat-related sequences will be important to clarify their evolutionary origins. The scale represents the number of substitutions per site.

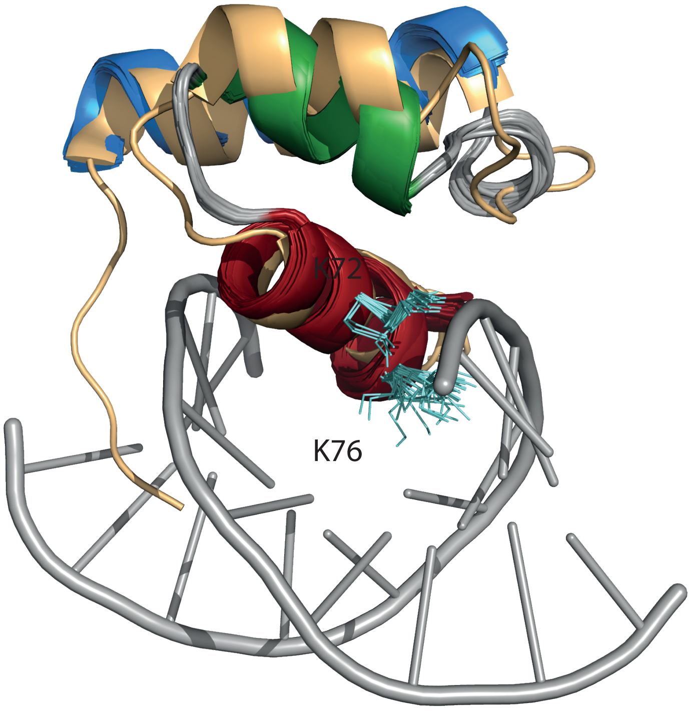

fig. S9. Model of a potential DNA binding mode of MatA. Overlaying the MatA NMR structural ensemble with the crystal structure (pdb 1APL) of *S. cerevisiae* MATα2 (light orange) bound to DNA (*27*) reveals residues that could potentially contact the DNA to coordinate the MatA-DNA interaction; the contributions of residues Lys-72 and Lys-76 (shown in turquoise) were assessed by testing mutants.

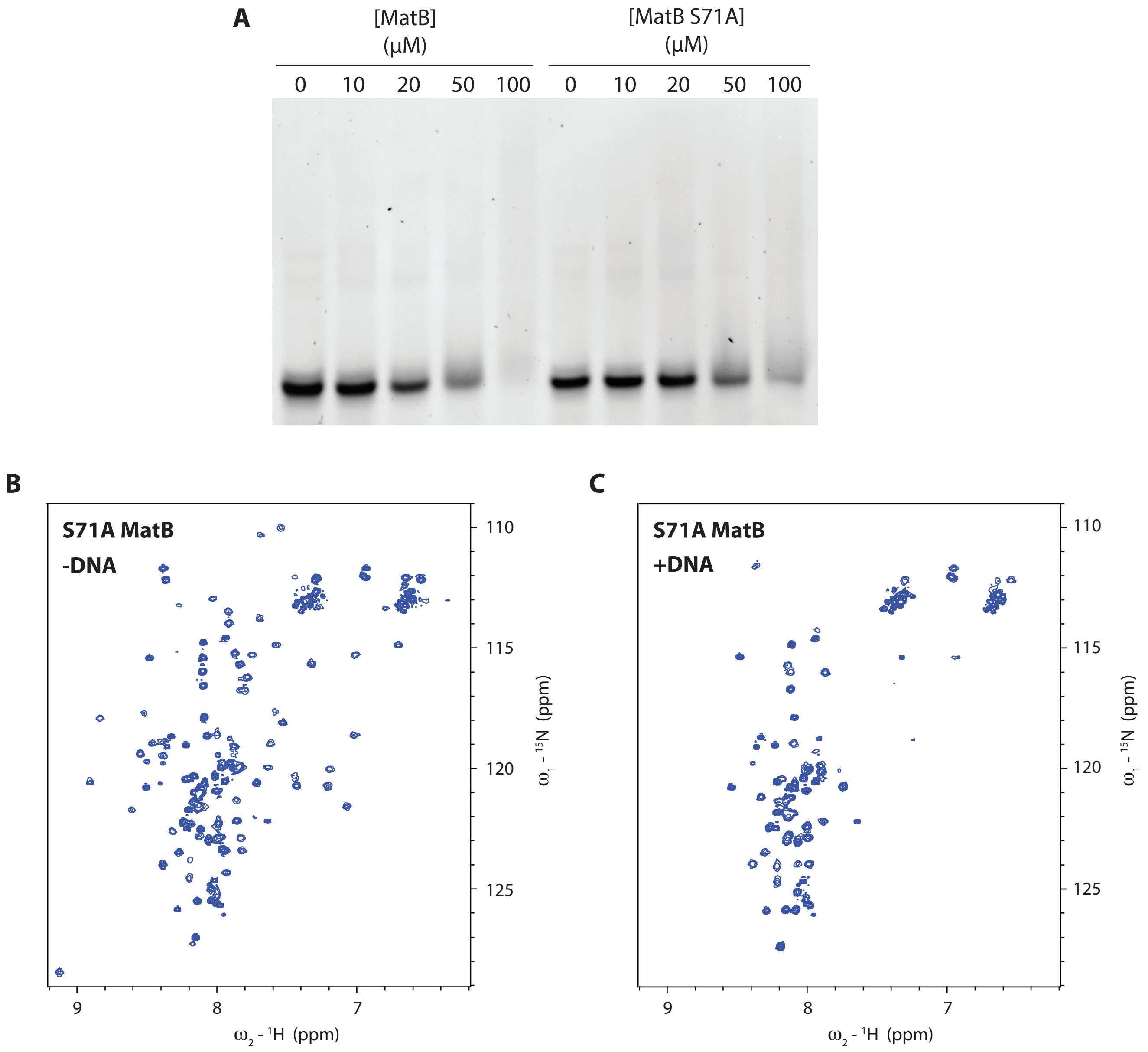

fig. S10. The DNA binding activity of MatB S71A. (**A**) EMSA experiments confirm that MatB S71A does bind, at least non-specifically, to double-stranded DNA. However, addition of DNA to a sample of MatB S71A in an NMR experiment (**B** and **C**) causes the loss of numerous peaks from throughout the folded core domain, likely due to line broadening caused by intermediate exchange between bound and unbound states of MatB. It was therefore not possible to characterise the DNA binding activity of MatB using this technique.

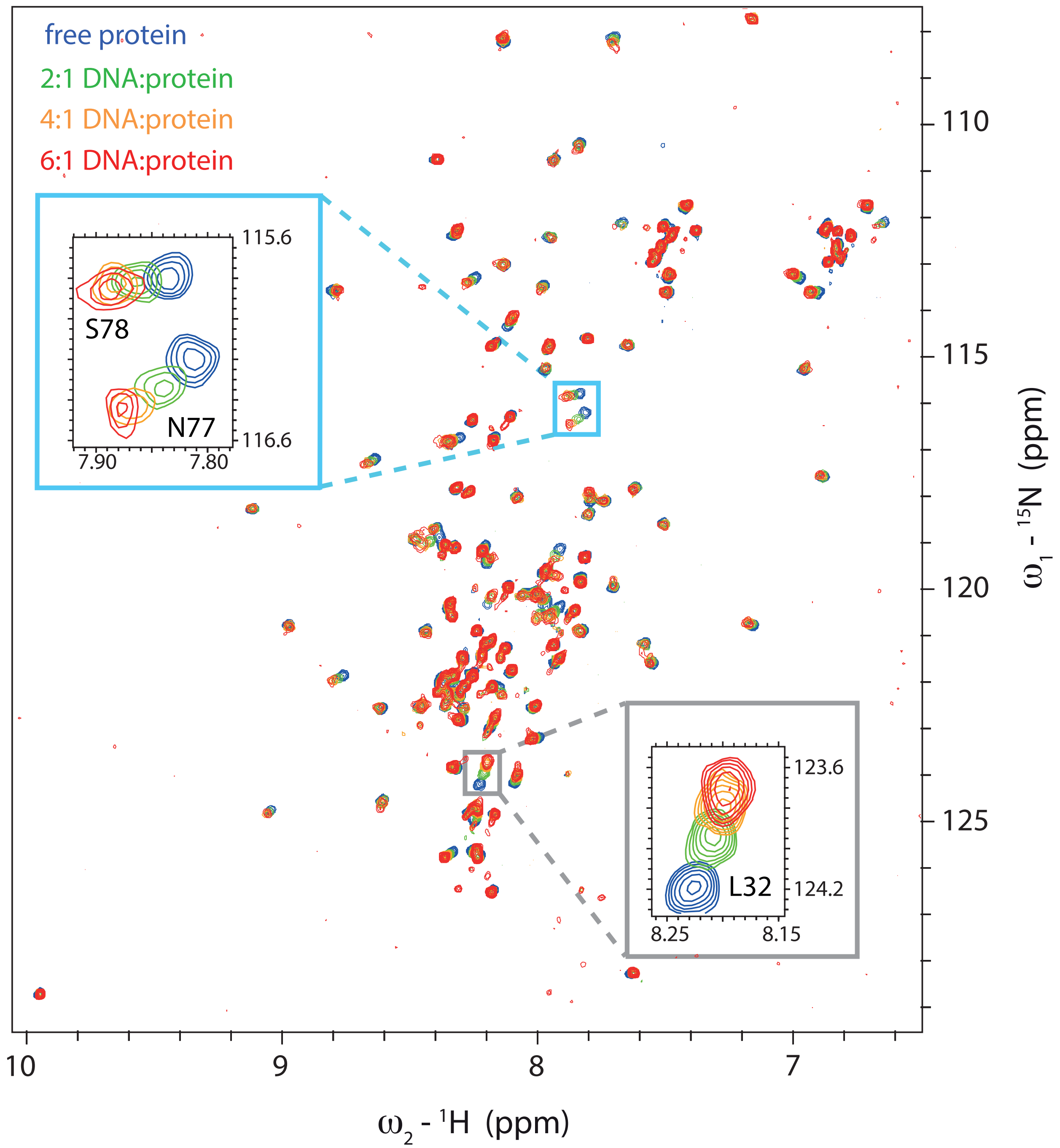

fig. S11. CSPs for MatA as a function of added 58-bp DNA concentration. Adding increasing amounts of the 58 bp DNA to MatA results in very similar chemical shift perturbations in the [^{15}N-^{1}H]-HSQC NMR spectrum as those seen as a function of increasing dsDNA length; because the binding to this non-cognate DNA is very weak, the protein remains unsaturated even at the highest DNA:protein ratio. These experiments employed MatA at 20 µM and 58 bp DNA at 40 µM, 80 µM and 120 µM, in 25mM phosphate pH 6.0, 50mM NaCl, 50 µM EDTA.

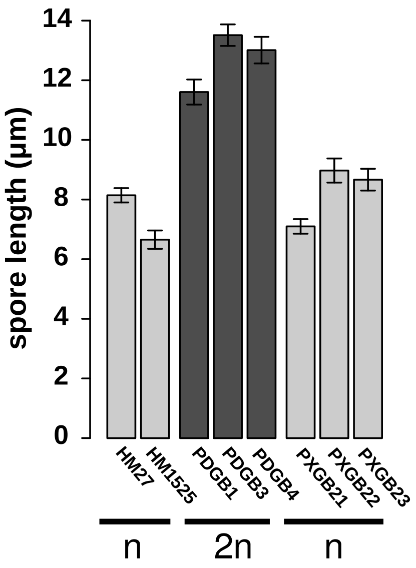

fig. S12. Spore size of haploid and parasexual diploid strains. Spores of parasexual diploids are larger than those of parental haploids, and segregant haploids have spores of similar size as the parental haploid strains. Fresh spores from the indicated strains were imaged using differential interference contrast microscopy and their length along their longest axis was measured. The mean ± SEM of nine spores for each strain is shown. Spores of HM27, PXGB22, and PXGB23 are more elongated than those of HM1525 and PXGB21, as is typical of strains derived from the V12 background (*75*).

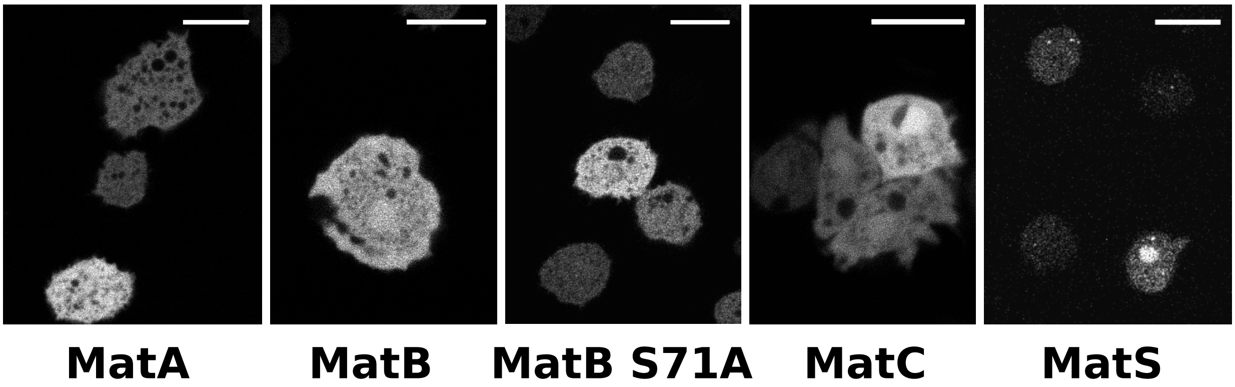

fig. S13. Localization of Mat proteins tagged with fluorescent proteins. The homeodomain-like folds and DNA-binding properties of MatA and MatB suggest that they function as transcription factors in the cell nucleus. When tagged with GFP and overexpressed, these proteins are distributed throughout the cytoplasm, with no evidence of exclusion from the nucleus, consistent with a nuclear function. We have not identified conditions in which these proteins localise to the nucleus preferentially compared with the cytosol. The S71A mutant of MatB behaves indistinguishably from wildtype MatB. Although the functions of MatC and MatS are not known, a parsimonious hypothesis is that they function cooperatively with MatA and MatB as transcriptional regulators. Although these proteins also tend to have evenly cytoplasmic conditions (with some suggestion that they form aggregates when overexpressed), when tagged with FusionRed (*76*), occasionally MatS has a localisation pattern consistent with nuclear enrichment.
